# Molecular Design Method Using a Reversible Tree Representation of Chemical Compounds and Deep Reinforcement Learning

**DOI:** 10.1021/acs.jcim.2c00366

**Published:** 2022-08-12

**Authors:** Ryuichiro Ishitani, Toshiki Kataoka, Kentaro Rikimaru

**Affiliations:** Preferred Networks, Inc., 1-6-1 Otemachi, Chiyoda-ku, Tokyo 100-0004, Japan

## Abstract

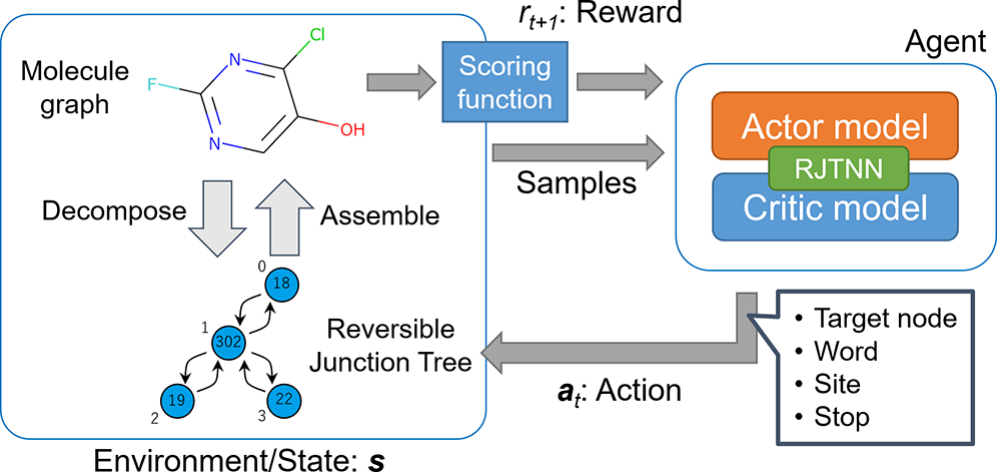

Automatic design of molecules with specific chemical and biochemical properties is an important process in material informatics and computational drug discovery. In this study, we designed a novel coarse-grained tree representation of molecules (Reversible Junction Tree; “RJT”) for the aforementioned purposes, which is reversely convertible to the original molecule without external information. By leveraging this representation, we further formulated the molecular design and optimization problem as a tree-structure construction using deep reinforcement learning (“RJT-RL”). In this method, all of the intermediate and final states of reinforcement learning are convertible to valid molecules, which could efficiently guide the optimization process in simple benchmark tasks. We further examined the multiobjective optimization and fine-tuning of the reinforcement learning models using RJT-RL, demonstrating the applicability of our method to more realistic tasks in drug discovery.

## Introduction

Exploration of novel chemical compounds is an important process in drug and material discovery. The process entails iterative trials, including the proposal of candidate compounds and their experimental evaluation, which are usually expensive and time-consuming. Furthermore, while the chemical space of feasible and chemically synthesizable compounds is astronomically large (estimated to be on the order of 10^60^), only a small portion of this space has been explored for drugs or functional materials. As it is impossible to explore compounds having the desired properties from this vast chemical space using *ad*-*hoc* experimental trials, computational exploration of chemical space has been proposed and is now gaining considerable attention owing to recent advancements in computational power. In computational exploration, all of the steps in the iterative trials, including the proposal of novel chemical compounds and their property evaluation, were performed *in silico*. Using extensive computational resources, this method extends the exploration range within the vast chemical space remarkably. However, owing to the discrete nature of chemical structure representation (*i.e*., graphs in discrete mathematics), the difficulty to efficiently explore the chemical space still persists.

To address this problem, new exploration methods using deep neural networks^1^ (DNNs; for comprehensive reviews see refs (2, 3)) have been proposed. One example is the latent-space-based method, which involves a variational autoencoder (VAE^4^). In this method, a DNN, known as a generative model, learns the mappings between the discrete space of the chemical compounds and the continuous latent manifold. After learning the generative model, exploration is performed over the continuous latent manifold.^5^ Bayesian optimization or metaheuristics-based methods were used for the optimization process because the molecular properties used as scoring functions are usually not differentiable with respect to the latent manifold, even though the latent space itself is continuous. However, this method has several limitations. The first concerns the dimensions of the latent manifold. For example, on using the practical size of the training dataset (10^5^–10^6^ compounds), learned latent manifolds with a reasonable reconstitution rate usually result in dimensions greater than 50, which is not easy for the available optimization algorithms. Second, no reliable indicator exists for assessing the rationality of learned mapping. Although we can evaluate the model using the reconstruction error against the training dataset, it is unclear whether a low reconstruction error is sufficient to guide the chemical structure optimization with the given scoring functions. The third problem is that the learning process of the generative model is detached from the optimization process of the chemical compounds with regard to the scoring function. The pretrained generative model remained unchanged during optimization and did not learn the scoring function. Recently, to address the third problem, the retraining of the generative model based on optimization results has been proposed.^6^ This method requires an iterative process of optimization and training, which is computationally expensive and time consuming.

Another example is a reinforcement learning (RL)-based method. In this method, the molecular design problem is formulated as a Markov decision process wherein an agent learns the optimal policy based on the rewards offered by its surrounding environment. The RL-based method can partly alleviate the problem of latent-space-based methods because the policy approximated by the DNN model learns the scoring function during optimization. RL-based methods can be classified into two types based on the representation of chemical compounds. The first type uses text representation (*e.g*., SMILES), which allows us to leverage techniques used in natural language processing. Although this representation is widely used (including in latent-space-based methods^5^), the generated text may contain grammatically invalid results that can affect optimization performance. This type of method includes the REINVENT.^7^ The second type uses a graph representation of the chemical structure. Examples of this type include GCPN^8^ and MolDQN.^9^ In this representation, the generated graphs are theoretically decodable to valid molecules, thereby avoiding the grammatically invalid results in the text representation. However, the molecular properties can change drastically even with a single action of RL, such as in the last step of closing a conjugated aromatic ring. This makes it difficult to guide the agent directly to the optimal action at each RL step. Thus, the molecular representation using chemical groups (*e.g*., phenyl groups) as building blocks seem effective for RL-based molecular design. In contrast, molecular structure generation algorithms using predefined fragments as building blocks have been continuously studied over the last century, and many approaches have been proposed.^10−15^ However, it is difficult to integrate such methods with learning-based optimization algorithms, including RL.

Recently, Jin et al.^16^ proposed a novel coarse-grained molecule representation by leveraging junction tree (JT) decomposition to a molecule graph. In this method, a molecule is represented as a tree (*i.e*., a graph without circular or closed paths) with nodes corresponding to rings or bonds. Similar to the all-atom graph representation, this JT representation always corresponds to a valid molecule by composing valid chemical fragments as building blocks. This method has been successfully applied to several molecular-generative models.^16,17^ However, the JT representation cannot be reversibly converted to the original molecule without auxiliary information. Therefore, additional neural networks (such as atom-based graph convolution networks) are required to supplement this information for decoding molecules from the generated JT representation, making this technique complicated and computationally intensive.

In this study, we propose a novel RL-based molecular generation and optimization framework that leverages JT-based representation. For this purpose, we tailored the JT representation to be reverse-convertible to the original molecule without auxiliary neural networks (reversible JT; RJT). This reversible molecular representation enabled us to formulate the molecular generation and optimization problem as tree-structure construction using RL (RJT-RL). The reversible nature of the RJT enables the evaluation of molecules decoded from the intermediate states in the RL episodes. We tested the method using simple molecular design tasks as benchmarks and subsequently applied it to more realistic tasks in drug discovery involving multiobjective scoring functions.

## Methods

### Reversible Tree Representation of Molecules

In the original JT representation, the molecular graph  is converted into a tree representation  using the JT algorithm.^16^ Briefly, the molecular graph  is fragmented into bonds and rings using the smallest set of smallest rings (SSSR) algorithm implemented in RDKit.^18^ Then, a node is assigned to each bond or ring, and the nodes are connected by an edge when they share atoms in  to construct the tree representation  (Figure S1). If the bond nodes intersect at more than one node, the resulting  creates a cyclic structure. This situation is avoided by inserting a single atom shared between these nodes as a “singleton node” (see the original study^16^ for a detailed description of the algorithm). Hereafter, we term the nodes representing the bond and ring as “bond nodes” and “ring nodes,” respectively. A possible combination of unique atom clusters in the dataset forms a vocabulary . Each node *N*_*i*_ is assigned a word ID *w*_*i*_ based on the atom cluster in the node. This coarse-grained representation  allows us to build a molecule from chemically valid fragments, thereby avoiding atom-by-atom molecular creation through chemically invalid intermediates. However, the JT representation  cannot be reversibly converted into the original molecule because the connection information between the nodes is lost in this coarse-grained representation. In previous studies,^16,17^ this problem was avoided by introducing supplementary neural networks, which complicated the JT representation framework.

**Figure 1:**
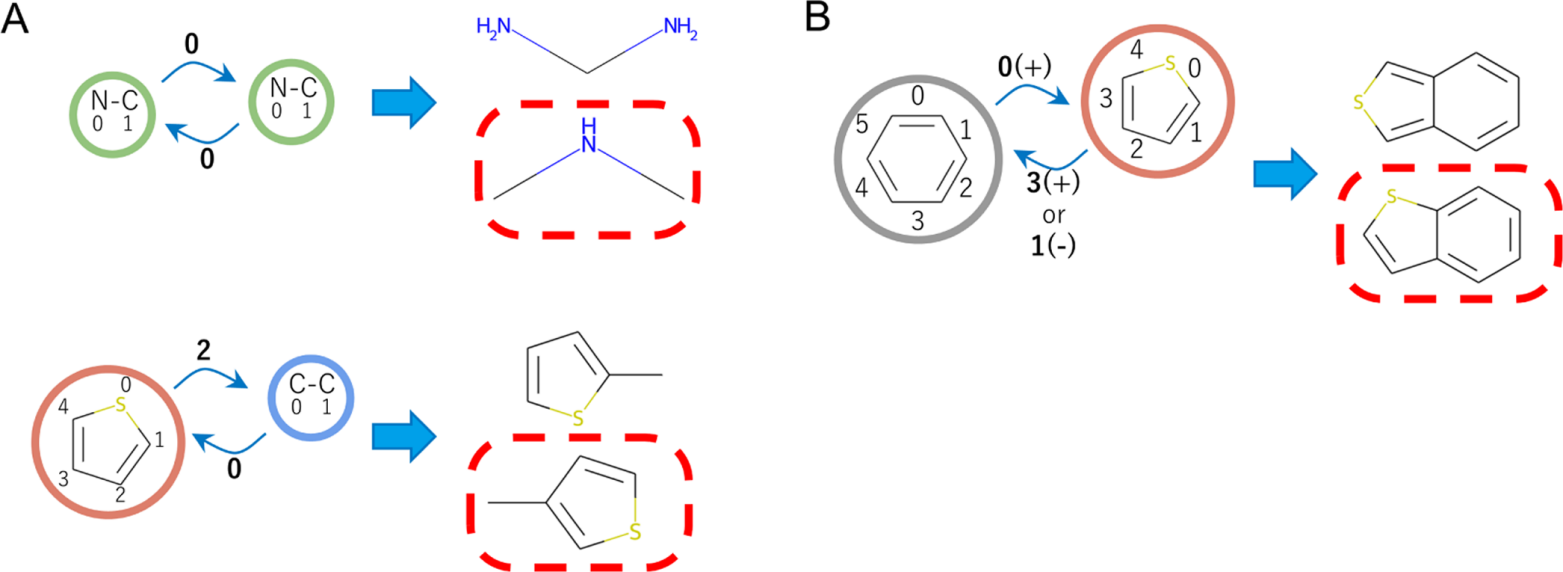
Definition of the site information that enables the reversible conversion to the original molecular structure. (A) Site information for type-1 edges. In the left panels, circles and arrows indicate the nodes and edges of the tree representation, respectively. Numbers near the arrows indicate site information. The left panels show the possible molecular structures assembled from the tree representation. Using site information, the original structure indicated by the dashed line can be selected without ambiguity. (B) Site information for type-2 edges. The numbers and (+)/(−) signs near the arrows indicate site information.

In this study, we introduce the reversible JT (RJT) representation () of molecules by extending the JT decomposition of . To eliminate arbitrariness in the node connection (*i.e*., to determine atoms shared between two nodes connected by an edge), we record the ID of the atoms shared between two adjacent nodes (*N*_*i*_ and *N*_*j*_) as the “site information” σ_*i*,*j*_. The site information is defined as follows (Figure 1). The original JT implementation involves sharing of one or two atoms between adjacent nodes connected by the JT edge. Hereafter, the JT edge sharing one atom is denoted as a “type-1 edge” (Figure 1A), while that sharing two atoms as a “type-2 edge” (Figure 1B). Type-1 edges can be further classified into three cases: edges connecting (i) bonds and singleton nodes, (ii) two bond nodes, and (iii) bond and ring nodes. In these three cases, the indices of the shared atom in both nodes are recorded as the site information σ_*i*,*j*_. In contrast, type-2 edges always connect two ring nodes, and the indices of the two atoms in both the ring nodes are recorded. Because the atoms shared between the nodes are always adjacently located in the ring, the indices of the first atom and the direction ID (+1 or −1) of the second atom are recorded as site information σ_*i*,*j*_. A spiro connection between two rings is treated as a special case of the type-2 edge by assigning a direction ID of 0, although only one atom is shared between the nodes. The most appropriate location for storing this site information is edge *E*_*i*,*j*_, which represents the existence of shared atoms in *N*_*i*_ and *N*_*j*_ in . Thus, in this study, the site information is encoded as edge features.

The algorithm for converting the RJT representation  to molecular graph  (Algorithm 1) can be described as follows: this method is similar to the assembly algorithm of Jin et al.,^16^ except that enumerating all combinations of node-to-node attachments in  is not necessary. In our method, the predicted  is traversed in the depth-first order by attaching a subgraph, corresponding to the child node, to the constructed graph. Using the site information, the atom(s) shared between two nodes can be uniquely determined, and the nodes could be deterministically assembled to convert  to . However, it is possible that the predicted site information is incompatible with the node types (*e.g*., more than four atoms are connected to one carbon atom). In such cases, the second (or third) probable site information can be utilized based on the output of the softmax logits of the neural network (described later). Alternatively, an exception could be raised to indicate that a particular RJT contains invalid information. The latter implementation was used to simplify the code.

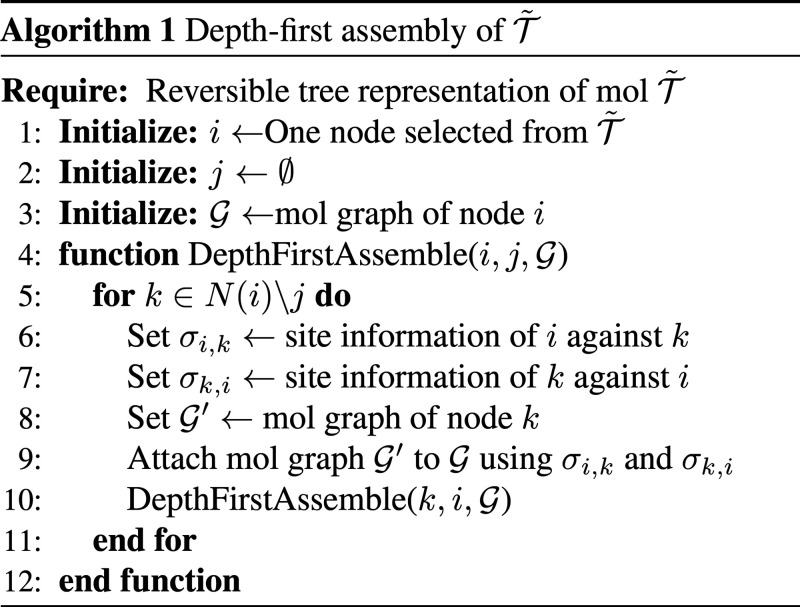



### RJT-Based Neural Network

The neural network architecture that encodes the RJT representation into hidden vectors with fixed sizes ({**h**_*i*_}) can be described as follows
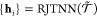


Each node *N*_*i*_ is represented by a one-hot vector of word ID *w*_*i*_, while edge *E*_*i*,*j*_ is represented by a one-hot vector of site information σ_*i*,*j*_. These one-hot representations of *w*_*i*_ and σ_*i*,*j*_ are then converted to node and edge features **x**_*i*_ and **y**_*i*,*j*_ using the learnable embedding matrices **E**^node^ and **E**^edge^, respectively, as follows
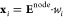

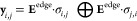


To encode the RJT, including both node and edge features, we extended the tree-based gated recurrent unit (GRU)^16^ as follows
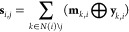

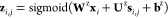

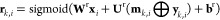

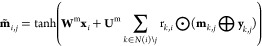

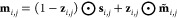


In the above formulas, we extended the tree-based GRU; however, the tree-based long short-term memory (LSTM)^17^ could also be extended to accommodate the edge features in a similar manner. The message vectors **m**_*i*,*j*_ are updated in two phases (bottom-up and top-down), as in the original tree-based GRU. Finally, the hidden vector **h**_*i*_ of node *N*_*i*_ is computed by aggregating the message vectors **m**_*i*,*j*_ as follows
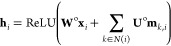
where ReLU stands for the rectified linear unit, defined as ReLU(*x*) = max(0, *x*).

### RL Using the RJT Representation

In this study, we formulated a molecular design task for tree generation using RL.^19^ In general RL settings, we consider that an agent receives state  from the environment and selects an action  according to its policy π = π(*a*_*t*_|*s*_*t*_) at each time step *t*, where  is a set of possible states and  is a set of possible actions at state *s*_*t*_.

After taking an action, the agent receives the next state *s*_*t* + 1_ and a scalar reward *r*_*t*_, and proceeds to the next step *t* + 1. The episode ends when the agent receives a terminal state at time step *T* and then proceeds to the next episode with an initial time step *t* = 0. The return *R*_*t*_ is the total accumulated reward from time step *t* to *T* with discount rate γ, as follows
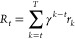


Action value *Q*^π^(*s*, *a*) is defined as the expected return on selecting action *a* in state *s* after following policy π, whereas the value of state *V*^π^(*s*) is defined as the expected return from state *s* after following policy π. The goal of the agent is to maximize the value of state *s*_*t*_, which is the expected return *R*_*t*_ from each state *s*_*t*_.

Policy-based RL methods directly parameterize the policy π_θ_ and optimize its parameter θ using the gradient estimator of . The policy gradient theorem states that the unbiased estimate of  can be estimated as^20^
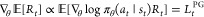


To reduce the variance of this gradient estimate, the estimate of advantage (*Â*_*t*_; *A*(*s*, *a*) = *Q*(*s*, *a*) – *V*(*s*)) was used instead of *R_t_*.^21,22^ Although the use of this advantage increases stability, it often leads to destructively large policy updates.

In the proximal policy optimization (PPO) algorithm,^23^ the clipped surrogate objective *L*_*t*_^CLIP^ is optimized instead of *L*_*t*_^PG^ to alleviate this problem as follows
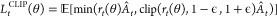
where *r*_*t*_(θ) = π_θ_(*a*_*t*_|*s*_*t*_)/(π_θ_old__(*a*_*t*_|*s*_*t*_)), and clip(•) clips *r*_*t*_(θ) outside the interval between [1 – ϵ, 1 + ϵ]. Then, the total loss function *L*_*t*_^PPO^, given as
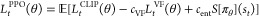
is optimized, where *L*_*t*_^VF^(θ) = (*V*_θ_(*s*_*t*_) – *V*_*t*_^targ^)^2^ is the squared error loss of the value function and *S*[π_θ_] (*s*_*t*_) is an entropy bonus term to ensure efficient exploration. For *Â*_*t*_, the truncated version of the generalized advantage estimation is used, as in the original PPO implementation, as follows
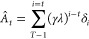
where δ_*i*_ = *r*_*t*_ + γ*V*(*s*_*t*+1_) –*V*(*s*_*t*_), and λ is the generalized advantage estimator parameter.^22^

### Definition of Policy and Value Function Networks

**Figure 2:**
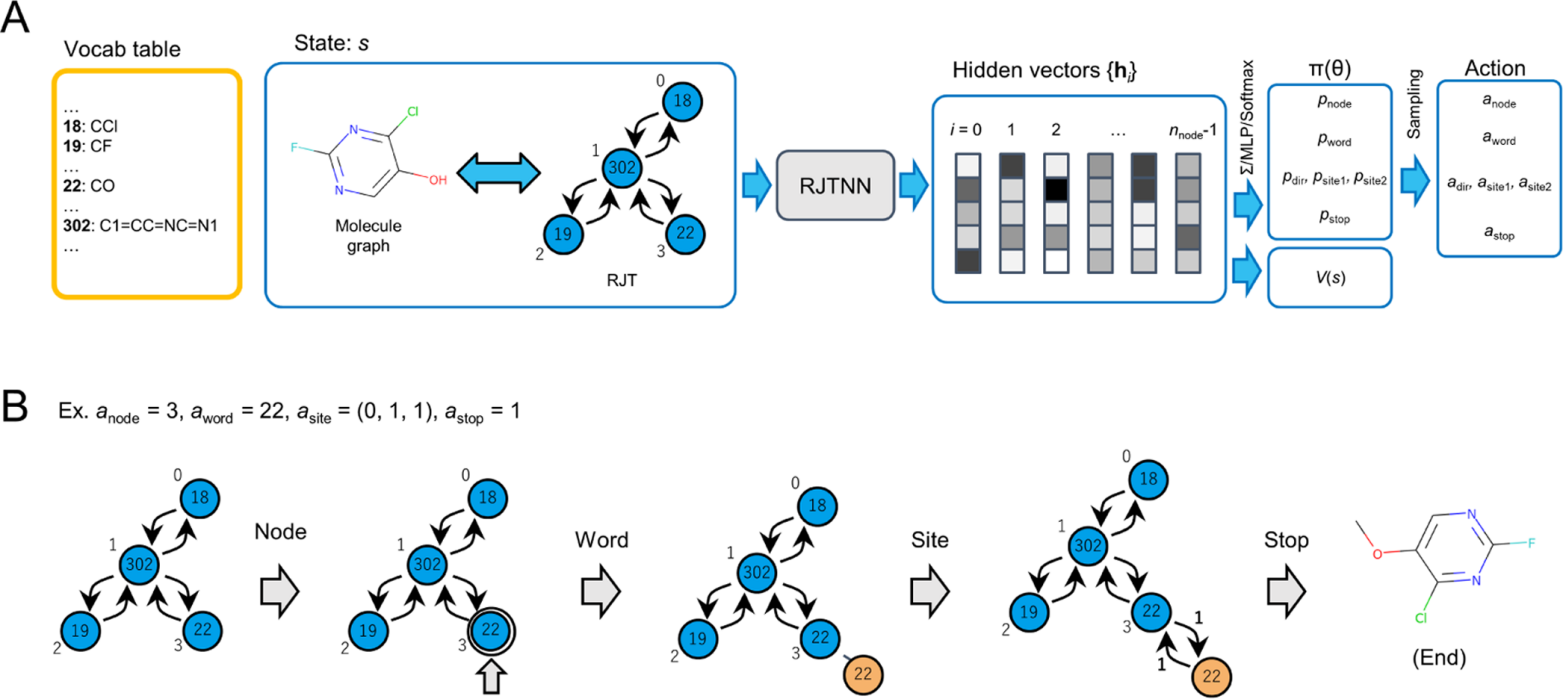
Network architecture of RJT-RL and the actions taken by the agent. (A) Calculation flow of the action and value function estimates. The RJT representing the state is converted to hidden vectors {**h**_*i*_} by the RJTNN network defined in the main text, using which the policy distribution π_θ_ and value function estimate *V*_θ_ are calculated using subsequent networks. The action taken by the agent is determined by sampling from this policy distribution. The numbers in the tree nodes indicate the word ID in the vocabulary table. (B) Example of an action sampled from the policy distribution and modification to an RJT representing the state. The state before the application of the action represents the molecule shown in panel (A). After applying the action, the state (*i.e*., RJT) is modified to represent the molecule shown in the leftmost panel.

The RJT representation of the molecule was used as the state  in RL, where  is a possible set of molecules that can be converted to RJT. The agent takes the action  to modify the RJT of *s*_*t*_ and constructs the RJT in the next step (Figure 2A). Here, the action *a*_*t*_ is defined using the following four components: (i) selection of the word ID *w*_*i*_ of the new node *N*_*i*_, (ii) selection of the existing node *N*_*j*_ for attaching to the new node, (iii) prediction of the site information σ_*i*,*j*_ (for connecting the two subgraphs represented by *N*_*i*_ and *N*_*j*_), and (iv) determining whether the episode ends (Figure 2B). The policy can then be expressed as a probability distribution function for each component of the action defined here. In this study, these probability distribution functions were approximated by RJTNN, using the RJT of *s*_*t*_ () as an input (Figure 2A).

First, we calculated the hidden vectors {**h**_*i*_} for each node of the RJT using RJTNN, as follows
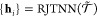


Then, each component of the policy distribution was calculated as follows
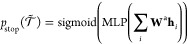

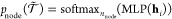

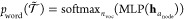

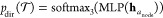

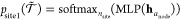

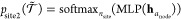
where *n*_node_ is the number of nodes in , *n*_voc_ is the size of vocabulary , and *n*_site_ is the maximum number of possible combinations of site information. The MLP function stands for a multilayer perceptron with two layers, including the ReLU activation function. The action (*a*_node_, *a*_word_, *a*_site_, *a*_stop_) taken by the agent is determined by sampling from this policy distribution as follows
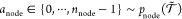

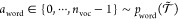

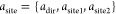

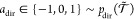

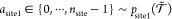

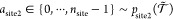

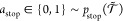


The value function is also approximated using the shared RJTNN with the policy network as follows
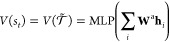


Using these neural network functions, the PPO target loss function (*L*_*t*_^PPO^(θ)) was minimized using the Adam optimizer during RL training. PyTorch^24^ and PFRL^25^ were used for the deep learning and RL frameworks, respectively, for the experiments.

### Expert Learning

To guide the exploration space of RL for drug-like molecules, the policy network was pretrained using the given dataset (“expert dataset”) similar to previous studies.^7,8^ For pretraining, the dataset molecules were converted to RJTs, and the root node was randomly selected from the sampled  from this dataset. The path for traversing  from the root node was generated in the breadth (or depth)-first order, from which one edge (connecting *N*_*i*_ and *N*_*j*_) was randomly selected. This edge represents the process of creating a new tree from a state comprising *N*_*i*_ and its ancestral nodes. Thus, the calculated state *s*_*t*_ and action *a*_*t*_ from the edge are used to minimize the negative log likelihood of the policy function, *i.e*.,
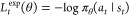


In the training phase of RL, the learned policy function is not expected to deviate significantly from the pretrained policy and generate non-drug-like molecules. The use of augmented likelihood in the REINVENT algorithm^7^ prevents the learned policy from deviating significantly from the pretrained model. In this study, we jointly trained the policy using an expert dataset with the target function of the PPO by minimizing the following target function
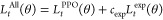
where coefficient *c*_exp_ is a hyperparameter that controls for the effect of the expert dataset.

### Experiment Settings

In all experiments, the valences of the atoms in the assembly process (conversion of RJT to a molecule) were checked. If excess valence was detected (*e.g*., a carbon atom with more than five bonds), a small negative score (*c*_invalid_) was given as the reward for step *t* to discourage the actions that produced invalid molecules. Next, in RJT-RL, any molecular property could be used as the reward function *r*_*t*_, which is calculated at every RL step because RJTs corresponding to intermediate states are convertible to valid molecules. Thus, we examined the effectiveness of this “step reward” and compared it with the “final reward” case.
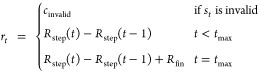
Here, *R*_step_ and *R*_fin_ are the step and final reward functions, respectively, defined according to the specific tasks, and *t*_max_ is the last time step in this episode. We also defined *R*_step_(−1) ≡ 0. Finally, the effect of the “duplication count penalty” on reward function was examined. One problem of RL is the balance between exploration and exploitation; poor exploration that maximizes short-term rewards results in trapping in the local minima. To avoid this problem, the entropy bonus term *S*[π_θ_](*s*_*t*_) was introduced into the loss function of the PPO.^23^ To further facilitate the exploration of the chemical space, we introduced a duplication count penalty function, which modifies the final reward *R*_fin_ calculated at the end of the episode as follows
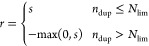
where *n*_dup_ is the duplication count of the generated molecule in this episode and *s* is the score calculated for the generated molecule. This function is intended to reset *R* (the total return of the episode) to zero if the duplication count exceeds a specified threshold. A threshold value of *N*_lim_ = 2 was utilized for all experiments in this study. This penalty term is inspired by previous studies,^26−28^ wherein the count-base bonus term was shown to facilitate exploration in various RL tasks.

All of the experiments described in the Results and Discussion section utilized the ethane molecule (“CC” in SMILES representation), which is the simplest atom cluster in vocabulary , unless otherwise stated. The policy network with a hidden vector size of 128 dimensions was pretrained using a dataset derived from the ZINC250k dataset.^5^ In the derived dataset, macrocyclic compounds or rings containing more than eight atoms were removed. Additionally, bridge compounds containing more than eight substitution sites were removed. The resulting dataset contains 246,416 molecules. We tested whether all RJT representations of the molecules in this dataset could be reversibly converted to the original molecules. For comparison, REINVENT,^7^ which is a SMILES-based molecular generator that uses RL, was used. The policy network was pretrained using the same dataset, and RL training was performed using the same scoring function and hyperparameters of *k* = 1 and σ = 20. In addition, CReM,^10^ a rule-based structure generation method using fragments as building blocks, was employed. The fragment library for CReM was generated using the ZINC250k dataset, and the optimization method described in this study was used for structure generation. Similar computational resources were used (CPU: one core of Intel Xeon Gold 6254 CPU@ 3.10GHz and GPU: NVIDIA Tesla V100) for all experiments.

## Results and Discussion

To examine the effectiveness of RJT-RL, we performed several experiments using molecular design as a benchmark. All of the experiments and their abbreviations are summarized in Tables 1, 2, and 3. We calculated several measures defined in the MOSES benchmark suite^29^ for molecules generated in the last 1/5 episodes.

**Table 1:** Summary of the Penalized Log *P* Experiments^a^

	method	reward	duplication penalty	Novel	Valid	Uniq	Frag	Scaf	IntDiv	Filt
P1	RJT-RL	step	off	1	1	0.991	0.078	0.016	0.593	0.602
P2	RJT-RL	final	on	1	1	1	0.132	0.024	0.521	0.807
P3	RJT-RL	step	on	1	1	0.996	0.077	0.021	0.593	0.553
P4	REINVENT			1	0.952	0.944	0.153	0.002	0.603	0.824

a The MOSES metrics^29^ were calculated for the molecules generated in the last 1000 episodes (Novel: novelty, Valid: validity, Uniq: uniqueness, Frag: fragment similarity, Scaf: scaffold similarity, IntDiv: internal diversity (*p* = 1), and Filt: fraction of molecules passing the unwanted structure filter).

**Table 2:** Summary of the Similarity Experiments^a^

	target	method	reward	duplication penalty	Novel	Valid	Uniq	Frag	Scaf	IntDiv	Filt
S1	vortioxetine	RJT-RL	step	off	1	1	0.069	0.169	0.027	0.348	0.543
S2	vortioxetine	RJT-RL	final	on	1	1	0.917	0.514	0.077	0.715	0.922
S3	vortioxetine	RJT-RL	step	on	1	1	0.849	0.245	0.002	0.641	0.829
S4	vortioxetine	REINVENT			1	0.970	0.970	0.970	0.689	0.837	0.693
S5	vortioxetine	CReM			1	1	1	0.608	0.034	0.724	0.488
C1	celecoxib	RJT-RL	step	off	1	1	0.100	0.119	0.098	0.387	0.982
C2	celecoxib	RJT-RL	final	on	1	1	0.951	0.183	0.011	0.776	0.809
C3	celecoxib	RJT-RL	step	on	1	1	0.926	0.357	0.004	0.745	0.777
C4	celecoxib	REINVENT			1	0.942	0.942	0.975	0.682	0.843	0.712
C5	celecoxib	CReM			1	1	1	0.608	0.034	0.724	0.488

a The metrics of the molecules generated during the last 4000 episodes were calculated, as shown in Table 1.

**Table 3:** Summary of the Docking Experiments^a^

	target	method	reward	3D conf	Novel	Valid	Uniq	Frag	Scaf	IntDiv	Filt
D1	interaction	RJT-RL	final	random	1	1	0.934	0.062	0	0.643	0.135
D2	interaction	RJT-RL	step	random	1	1	0.875	0.374	0	0.682	0.096
D3	interaction	RJT-RL	final	Enum	1	1	0.845	0.627	0	0.731	0.333
D4	interaction	CReM		random	1	1	0.999	0.580	0	0.562	0.378
M1	multiobjective	RJT-RL	final/step	random	1	1	0.933	0.176	0.002	0.721	0.950
M2	multiobjective	CReM		random	1	1	1	0.803	0	0.640	0.586
F1	fine-tuning	RJT-RL	final/step	random	1	1	0.876	0.147	0	0.685	0.458

a The metrics of the molecules generated by the last 10,000, 8000, and 2000 episodes for D1–D4, M1–M2, and F1, respectively, were calculated, as shown in Table 1.

### Penalized Log *P* Optimization

First, we verified the effectiveness of our method using a single chemical property that is easily calculated using only chemical structure. In the first experimental setup, we evaluated the penalized Log *P* score,^30^ which is the water–octanol partition coefficient (Log *P*) that also accounts for the ring size and synthetic accessibility (SA) score.^31^ Although the maximization of the penalized Log *P* score itself has no actual application in drug discovery, we first attempted this task to assess whether our method can optimize the computationally easy problem beyond the example molecules in the training dataset. Furthermore, many previous studies that performed optimization in the chemical space have employed this target score, thereby allowing the comparison of our method with these studies. For RJT-RL, we examined three cases (P1, P2, and P3 in Table 1) that differed in the settings of the reward evaluation (step or final) and the duplication penalty. In P1 and P3, the penalized Log *P* score was used as the step reward function, *R*_step_, but the duplication penalty was turned off in P1. In P2, the penalized Log *P* score was used as *R*_fin_, and no reward was given for the intermediate steps.

**Figure 3:**
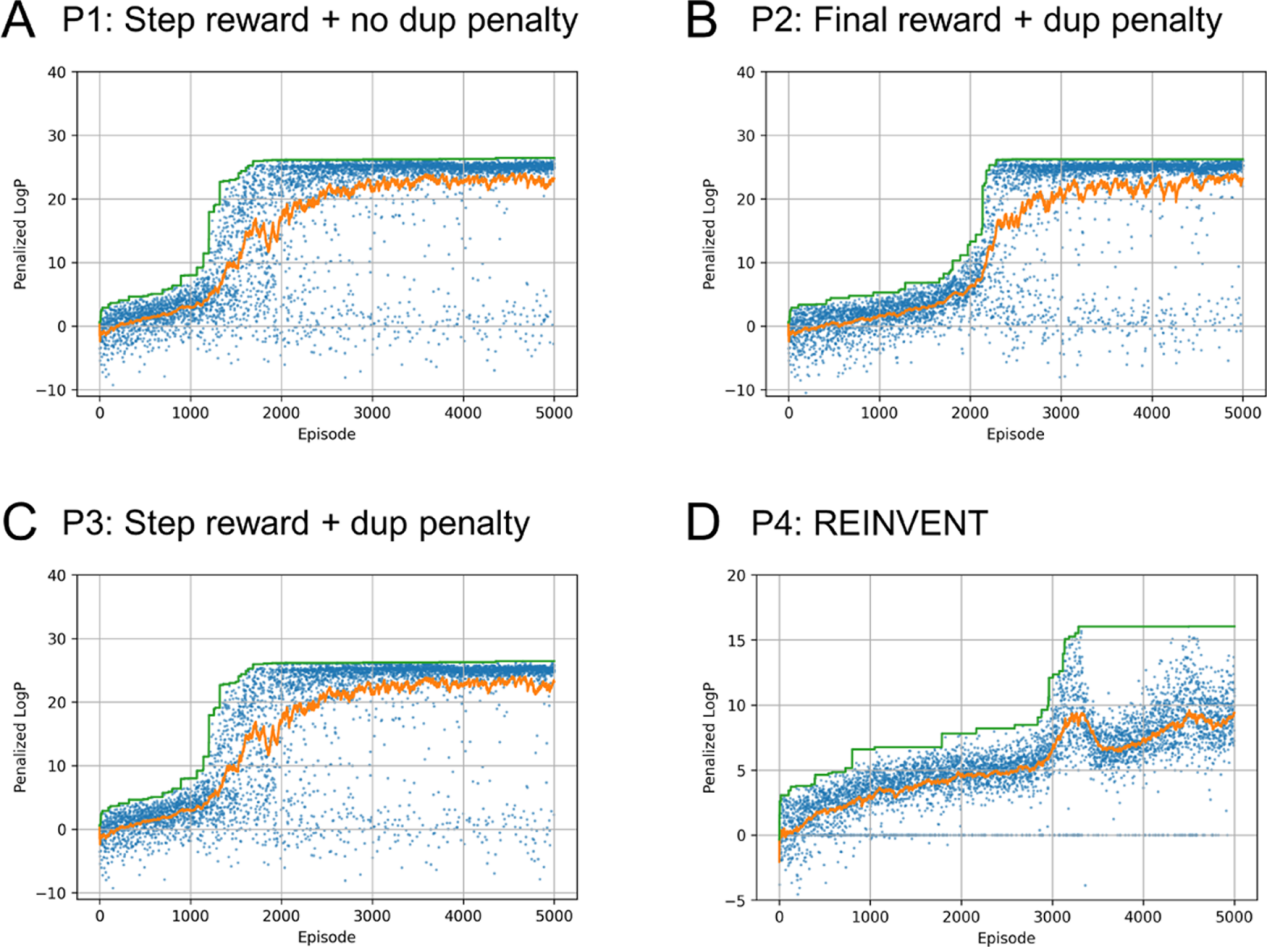
Results of penalized Log *P* optimization by (A–C) RJT-RL (P1–P3; see Table 1) and (D) REINVENT. The penalized Log *P* values for each episode are plotted in blue, whereas their moving average and maximum values are plotted in orange and green, respectively.

The results are shown in Figures 3, and S2 and Table 1. In all cases (P1, P2, and P3), molecules with high rewards were continuously generated, even in the absence of the duplication penalty (Figure 3A–C). Diverse molecules were generated without any penalty (uniqueness in Table 1); thus, the duplication penalty effect may be limited in this experimental setup. The comparison of the final and step reward cases (P2 *vs* P1/P3) showed that the reward functions tended to rise faster in P1/P3 than in P2 (Figure 3A–C). A similar tendency was observed in all other experiments, starting from different random states (Figure S3). These results suggest that the rewards of the RL intermediate states facilitated the optimization process. The REINVENT model (P4) also continuously generated high-scoring molecules, but the scores did not exceed those generated by our method (Figure 3D). The top three penalized Log *P* scores obtained in the experiments are summarized in Table 4, along with the results from previous studies. The comparison shows that our method performed comparably to the weighted retraining method,^6^ which also updates the generative model during the optimization process according to the target function.

**Table 4:** Summary of the Results of the Penalized Log *P* Optimization Experiment Performed in this and Previous Studies^a^

			best scores		
method	reward type	duplication penalty	1	2	3
RJT-RL (P1)	step	off	26.45	26.39	26.34
RJT-RL (P2)	final	on	26.23	26.22	26.21
RJT-RL (P3)	step	on	26.45	26.39	26.34
REINVENT (P4)			16.04	16.03	15.71
CharVAE			1.98	1.42	1.19
JT-VAE			5.3	4.93	4.49
GCPN			7.98	7.85	7.80
MolDQN			11.71	11.63	11.63
Weighted retraining			27.84	27.59	27.21

a Previous studies include the Following Methods: CharVAE,^5^ JT-VAE,^16^ GCPN,^8^ MolDQN,^9^ and Weighted Retraining^6^

### Similarity-Guided Molecule Generation

Next, we verified the effectiveness of our method using a simple task to generate molecules similar to a given query structure. To measure the similarity between molecules *i* and *j*, we used the Jaccard index^32^*J*_*i*,*j*_ of the RDKit implementation of the FCFP4 fingerprints.^33^ Although the similarity-guided optimization problem could seem trivial to human intuition, this task is significant for computational optimization using fingerprints as the similarity measure. For example, when attempting to generate an ether group, the alcohol moiety must be generated before reaching the final state. The fingerprint bits of alcohol and ether oxygens are different in the definition of FCFP4;^33^ the optimization target score decreases in the step generating the intermediate alcohol moiety. Therefore, the landscape of the fingerprint-based similarity score is not smooth but contains many local minima wherein simple optimization algorithms can get trapped. This local-minima problem of fingerprint similarity could be avoided using the final reward (*i.e*., evaluating only the final molecule in the episodes). However, this makes it impossible to evaluate each RL state value in the episodes. To evaluate the effectiveness of different reward settings, we examined three different cases of RJT-RL (Table 2).

#### Vortioxetine Rediscovery Experiment

**Figure 4:**
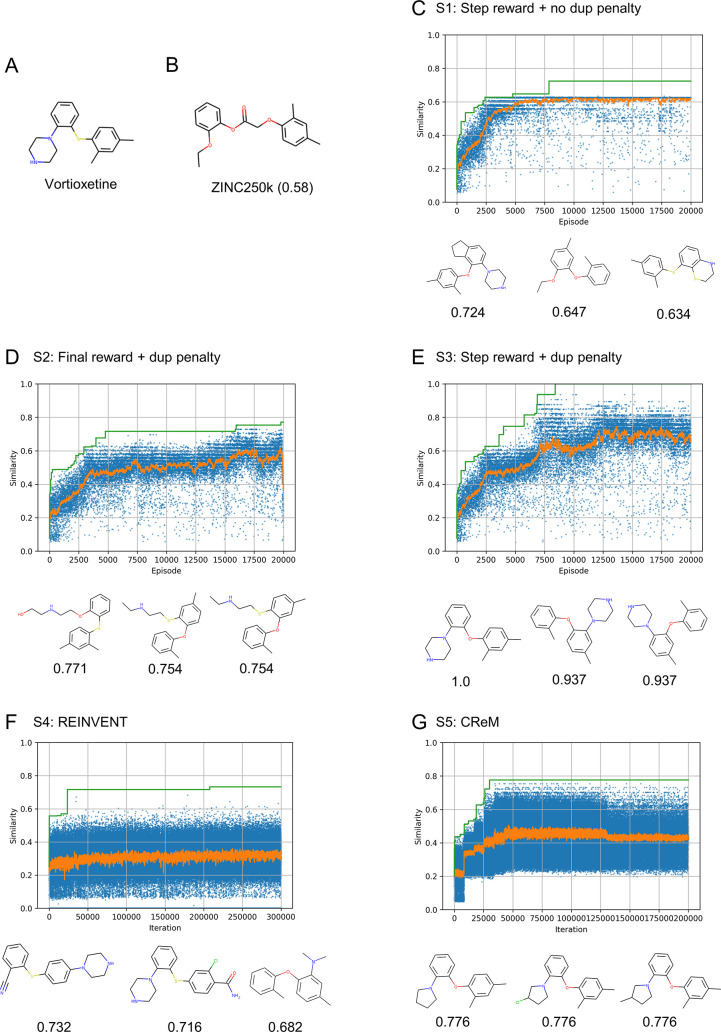
Compounds generated by the similarity optimization experiment targeting vortioxetine. (A) Chemical structure of vortioxetine used in the query structure in the experiment. (B) Compound most similar to vortioxetine in the ZINC250k dataset with its similarity score in parentheses. (C–G) Results of similarity optimization experiments, S1–S5 (see Table 2). The similarity scores for each episode (or iteration) are plotted in blue, and the moving average and maximum values are plotted in orange and green, respectively. The chemical structures of compounds with the highest scores (top three) are shown. Similarity scores are noted below the chemical structures.

In the first experiment (S1–S5; Table 2), vortioxetine was used as the query structure (Figure 4A). The most similar molecule in the pretraining dataset is shown in Figure 4B, with a similarity of 0.58. The results of the vortioxetine rediscovery experiment are summarized in Figures 4C–G and S4. In case S1, optimization was performed with a step reward and without a duplication penalty (Table 2), thereby achieving a similarity score of 0.724 (Figure 4C). However, after convergence to a local minimum, the exploration efficiency suddenly deteriorated, and similar or the same molecules were repeatedly generated (uniqueness in Table 2 and Figure S4A). In case S2, when only the final reward and duplication penalty were applied (Table 2), molecules with a similarity score of approximately 0.5 were continuously generated (Figure 4D), and a highest similarity of 0.771 was achieved (Figure 4D). In case S3, with the application of step reward and duplication penalty, diverse molecules with high similarity scores were continuously generated (Figure 4E). The average similarity score between the last 1000 episodes was 0.681, and a structure that was almost identical to the query structure was generated (Figure 4E). The best and average performances for S3 outperformed those for S2, demonstrating that the step-by-step evaluation of the molecular properties successfully guided the generation of optimum molecules. Additionally, several runs were conducted with different random seeds for S1–S3 (Figure S5), and it was observed that the duplication penalty strongly facilitated optimization in all cases. The step reward (S3) tends to produce better results than the final reward (S2), but it is influenced by random states to some extent. These results suggest that the advantage of step reward outweighs the disadvantage arising from the local minima in this problem.

In S4, REINVENT also generated structures that were similar, but not identical, to the query structure, with the highest similarity score of 0.732 (Figure 4F). However, structures with high rewards were generated infrequently, and an average similarity score of approximately 0.32 was obtained at the end of training (Figure 4F). This suggested that the agent failed to adequately explore the chemical space around the query structure. In S5, CReM also generated similar structures, with the highest similarity score of 0.776 (Figure 4G). The average similarity score at the end of the optimization was approximately 0.5 (Figure 4G). However, it requires many evaluations (approximately 50,000) of the score function as compared to the other experiments (approximately 10,000).

#### Celecoxib Rediscovery Experiment

**Figure 5:**
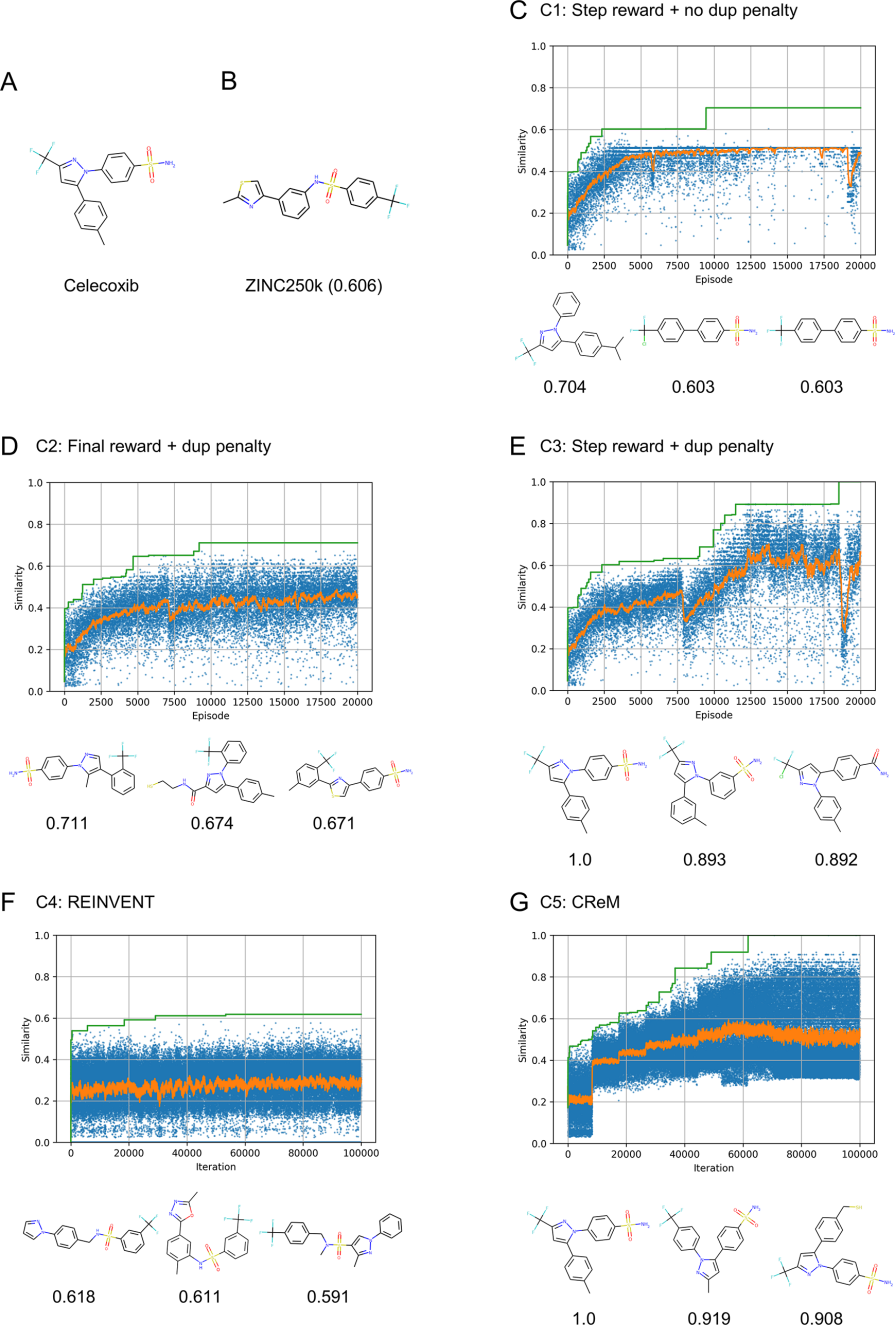
Compounds generated by the similarity optimization experiment targeting celecoxib. (A) Chemical structure of celecoxib used in the query structure of the experiment. (B) Compound most similar to celecoxib in the ZINC250k dataset with its similarity score in parentheses. (C–G) Results of the similarity optimization experiments, C1–C5 (see Table 2). The similarity scores for each episode (or iteration) are plotted in blue, and the moving average and maximum values are plotted in orange and green, respectively. The chemical structures of compounds with the highest scores (top three) are shown. Similarity scores are noted below the chemical structures.

Next, similarity-guided optimization of celecoxib, which is also included in the GuacaMol benchmark suite,^34^ was performed to observe the effects of the query structures (Figure 5A). For the experiment, RJT-RL, REINVENT, and CReM were used under conditions similar to those for vortioxetine (C1–C5; Table 3). The molecule most similar to celecoxib in the pretraining dataset is shown in Figure 5B, with a similarity of 0.606.

The results of the experiment are summarized in Figures 5C–G and S6. In RJT-RL, C3 (step reward) performed better than C1 and C2, as observed in the vortioxetine experiments (S1–S3). C3 generated a structure identical to celecoxib with a similarity of 1.0, but some effect of the random state on the generated structures was observed (Figure S7). CReM (C5) also successfully generated a structure identical to celecoxib after optimization of the 8th generation with an evaluation of approximately 60,000 compounds (Figure 5G). This result suggests that CReM is also a powerful method but requires a large number of score evaluations. In contrast, the best molecule generated by REINVENT (C4) has a similarity of 0.62 (Figure 5F), which is slightly better than the most similar structure in the dataset (Figure 5B). The original paper on REINVENT^7^ stated that it generated an identical structure to celecoxib under similar conditions but with a larger size (1.5 million) of the pretraining dataset. REINVENT may require a larger pretraining dataset to achieve the best performance.

### Structure-Based Scaffold Hopping

**Figure 6:**
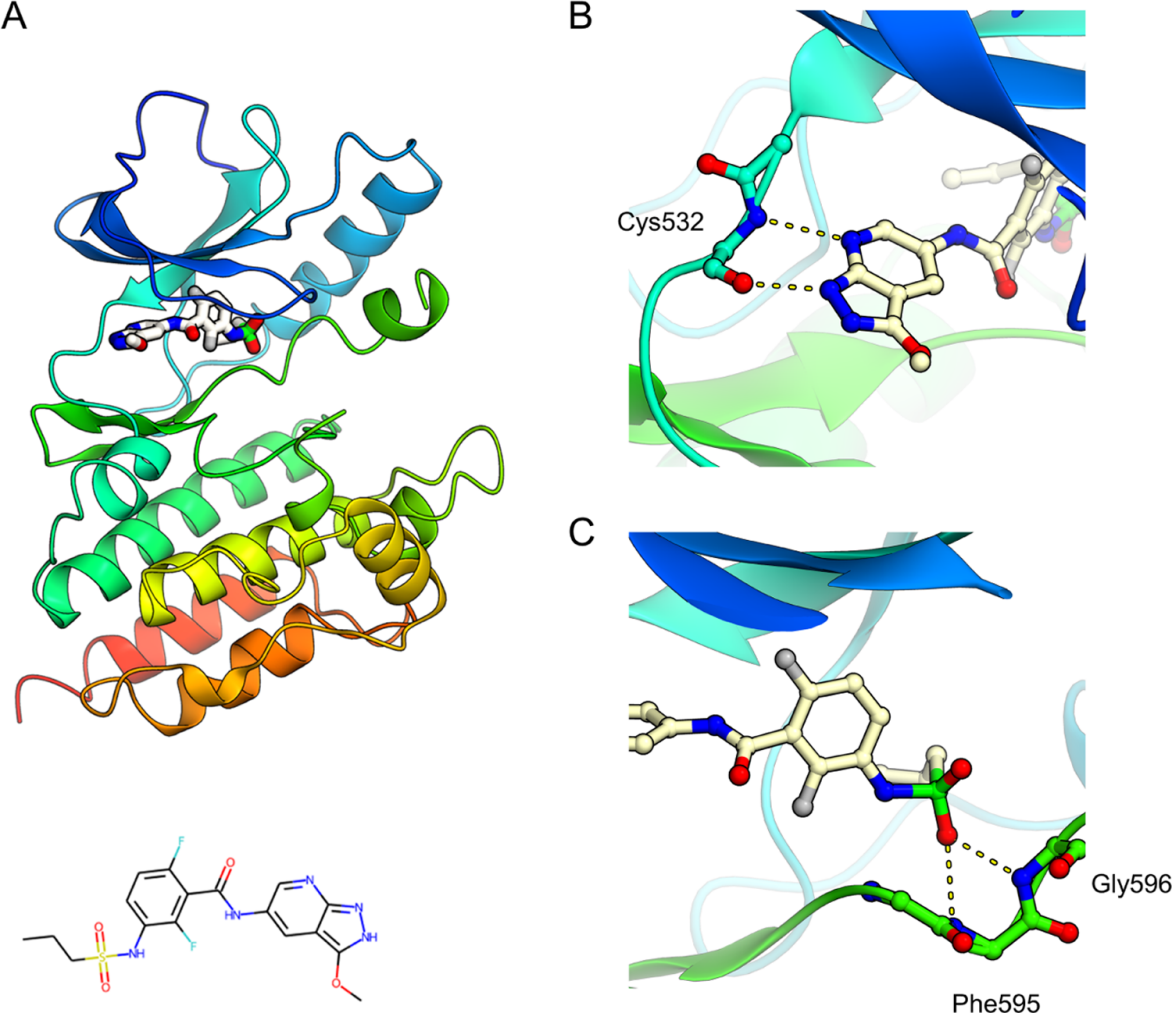
B-Raf kinase with its inhibitor complex^35^ (PDB ID: 3TV6) was used as the target structure in the structure-based scaffold-hopping experiments. (A) Overall structure, (B) interactions between the kinase hinge motif and the inhibitor, and (C) interactions around the sulfonamide moiety of the inhibitor. The protein backbone and bound inhibitor are presented using ribbon and ball-and-stick models, respectively. Molecular graphics were prepared using CueMol (http://www.cuemol.org/).

We further examined the efficiency of RJT-RL in the realistic task of structure-based scaffold hopping, wherein known drug candidate compounds with structural information (*i.e*., important interactions between the target protein and compounds) are available. B-Raf kinase with its inhibitor complex structure^35^ (PDB ID: 3TV6, Figure 6A) was used as an example. In this structure, the pyrimidine nitrogen of the inhibitor hydrogen bonds with the main-chain nitrogen atom of Gly 596 (Figure 6B) in the kinase hinge region. In addition to this hinge interaction, this inhibitor interacts with the main-chain nitrogen atom of Cys 532 through its sulfonamide group (Figure 6C). In practical structure-based drug discovery applications, it is crucial to search for molecules with different scaffolds that retain known important interactions with target proteins. Thus, we attempted to design novel compounds that preserved the interaction between the hinge region of the enzyme and the pyrimidine group in the compounds while changing the scaffold in the compound interacting with Cys 532.

In this task (D1–D4; Table 3), we used the docking score and the interaction score pertaining to the hydrogen-bond acceptor of the compound and the main-chain nitrogen atoms of Gly 596 and Cys 532. The ETKDG algorithm implemented in RDKit was used to generate three-dimensional (3D) conformations from two-dimensional (2D) structures.^36^ In this process, we randomly selected one stereoisomer from all possible stereoisomers except for D3, wherein we enumerated all possible stereoisomers up to 16, attempted docking simulations, and then selected the isomer with the best docking score. The interaction score is defined as follows
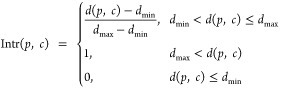
where *d*(*p*, *c*) is the distance between the specified atoms of the protein and the compound. In this experiment, *d*_max_ = 6.0 Å and *d*_min_ = 3.0 Å were used. The total reward function is defined as follows
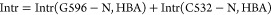

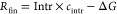
where HBA is the hydrogen-bond acceptor atom in the compound, Δ*G* is the AutoDock Vina score^37^ of the docking pose of the compound, and *c*_intr_ is a hyperparameter. Given the scale of the typical docking score (around −10) and interaction score (0–1) terms, *c*_intr_ = 10.0 was used in this experiment. The docking score was clipped by 500 because the PPO algorithm was destabilized by an extremely high value of the docking score. The pyrimidine group was used as the initial state for the RL training. The initial binding pose of the pyrimidine group to the protein was restrained to the same as that in the crystal structure. Two types of reward functions were compared as in the other experiments (Table 3). In cases D1 and D3, the aforementioned reward function was used for the final step, while *R*_step_(*t*) = 0 was maintained for all other intermediate steps. In case D2, the same function was used for *R*_step_(*t*). Furthermore, for comparison with the rule-based methods, CReM was examined using the same scoring function, including the docking and interaction scores (D4; Table 3).

**Figure 7:**
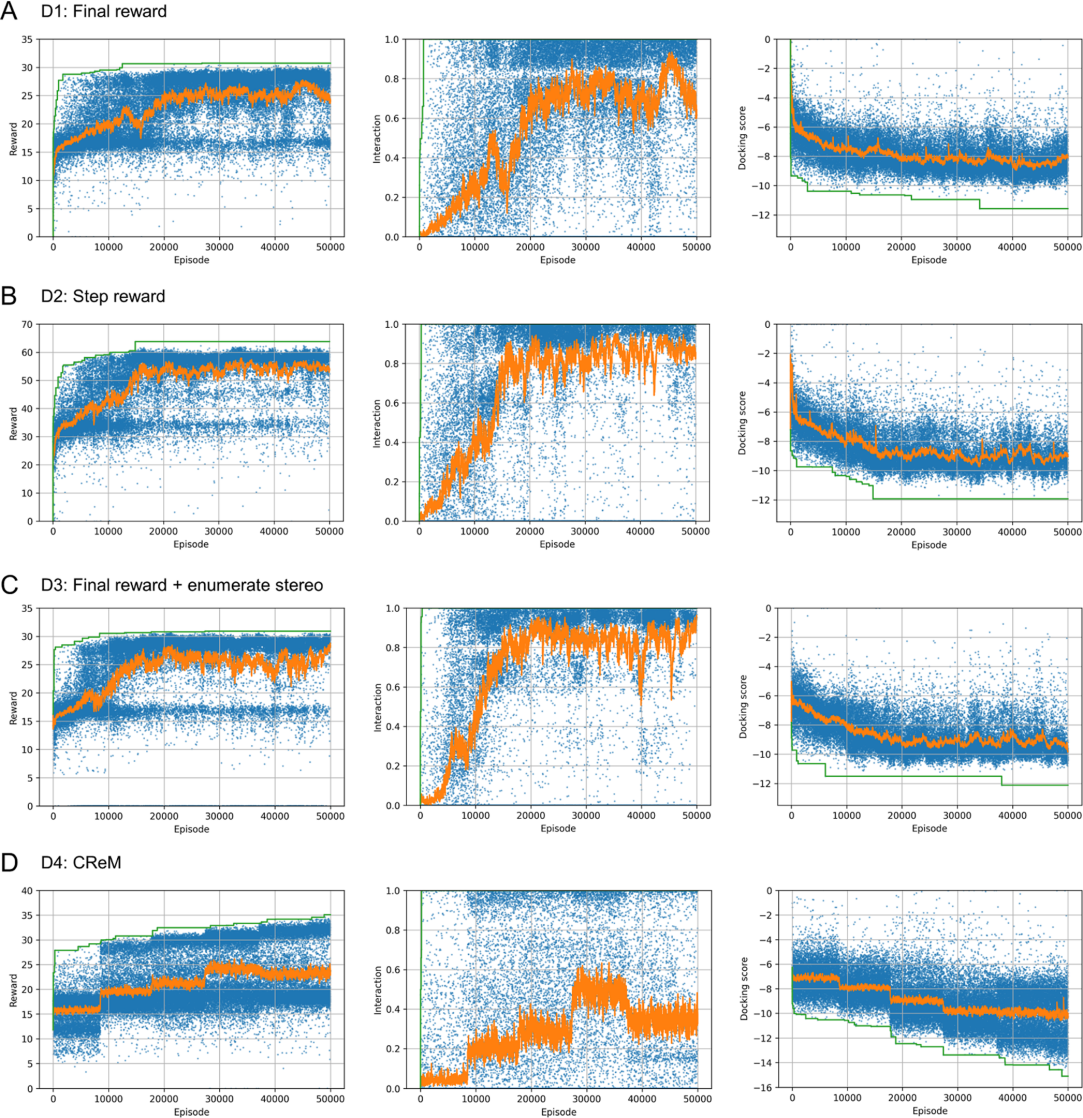
Results of the structure-based scaffold-hopping experiments, D1–D4 (see Table 3). The reward (left panel), interaction (middle panel), and docking (right panel) scores for each episode are plotted in blue, and the moving average and maximum values are plotted in orange and green, respectively.

The results for D1–D3 demonstrated that RJT-RL successfully generated optimized molecules with the desired interactions (Figure 7A–C). Both the interaction and docking scores were gradually optimized during the RL training episodes, and high-score compounds were continuously sampled during the later training episode stages. However, the distributions of the Log *P*, SA, and QED^38^ scores of the generated compounds tended to deviate from those of the training dataset (Figure S8A–C). Compounds with high docking scores that satisfied the desired interactions are shown in Figure S9. Next, we compared the results of the final and step rewards (D1 and D2, respectively). The step reward was marginally better than the final reward. D2 obtained a higher reward than D1 for the same number of episodes and converged to the best score faster than D1 (Figure 7A,B). This result also demonstrates the potential of the step-by-step evaluation of the molecular properties. However, the speed of D2 was much lower than that of D1 because the docking simulation was performed for each RL step in D2. Notably, D2 took approximately seven times more time than D1 to reach the same number of episodes (Table 5).

**Table 5:** Average Computational Time Required for Generating One Molecule

	reward	3D conf	average time (s)
D1	final	random	1.74
D2	step	random	12.9
D3	final	enum	8.96

In D3, we enumerated all stereoisomers, selected the one with the best docking score, and used the final reward function. This result demonstrates that the enumeration of stereoisomer accelerated the speed of optimization compared to that of D1 (Figure 7C). However, the speed of D3 was approximately four times lower than that of D1, as it performed up to 16 docking simulations per episode (Table 5). In D1 and D2, the randomly determined stereoisomers in the 3D-embedding process may result in different docking scores even though the agent performed the same actions. This random selection of stereoisomers may affect the optimization speed.

Next, we compared the results for RJT-RL (D1) and CReM (D4). Because the score evaluation, including the docking simulation, is a bottleneck in this task, we considered one score evaluation in CReM corresponding to one episode in RJT-RL with a final reward. The results show that the generated molecules are optimized against the target score function at a speed comparable to that of D1 (Figure 7D). However, the distribution of each score term is quite different from that of RJT-RL (D1; Figure 7A). In D3, the docking score term is well optimized, while the interaction term is less optimized than in D1 (Figure 7D). One possible reason for this unbalanced optimization is the tendency of CReM to generate large molecules (Figures S8D and S9D). This tendency may increase the van der Waals interaction terms in the Vina scoring function,^37^ thereby causing an unbalanced optimization. This could be avoided by optimizing *c*_intr_, the balancing hyperparameter of the score terms, or using the ligand efficiency^39^ instead of the docking score.

Finally, we examined several runs with different random seeds for D1, D2, and D3 (Figure S10). In all cases, the optimization proceeds similarly, but the convergence speeds differ depending on the runs in D1 and D2. In particular, in another run of D2, the speed of convergence is observed to be similar to that of D1 (Figure S10B). Next, we examined the differences in the molecules generated from different runs. We calculated the pairwise similarities of the top 100 molecules from the two runs and plotted their distributions (Figure S10D). The results demonstrate that diverse molecules are generated from different runs with the same hyperparameters. This could be an advantage of RJT-RL because it is important to obtain a variety of compounds with high target scores in actual drug design tasks. Consequently, given the level of improvement and deterioration by the step reward (D2) and stereoisomer enumeration (D3), we conclude that the final reward (D1) is best under the current experimental settings.

### Multiobjective Optimization

While the above cases targeted simple rewards with one or two objectives, a typical drug design task requires the improvement of multiple objective functions. In particular, the compounds generated in cases D1–D3 tended to contain highly hydrophobic and/or possibly unstable structures (Figures S8A–D and S9), as also evident from the low “filter” score of D1–D3 in Table 3. These unfeasible structures can be suppressed using multiobjective target functions, including penalties for unwanted structures. In M1, we introduced SA^31^ and Log *P* scores to penalize such unfeasible structures and examined the performance of our method in multiobjective optimization tasks. We define two penalty terms as follows.
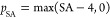

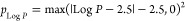
where SA and Log *P* are the SA and Log *P* scores, respectively, of the target molecule. Thus, these terms are intended to restrict the SA score to less than 4 and the Log *P* score to the range of 0–5. The total reward function is defined as follows
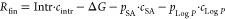

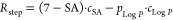
where *c*_intr_ = 10, *c*_SA_ = 1, and *c*_LogP_ = 5 were used. We standardized and neutralized the compounds using the functions implemented in RDKit^18^ before generating the 3D conformations. Additionally, we examined the CReM generator with similar settings, except for the target score function, which is defined as *R*_fin_ above.

**Figure 8:**
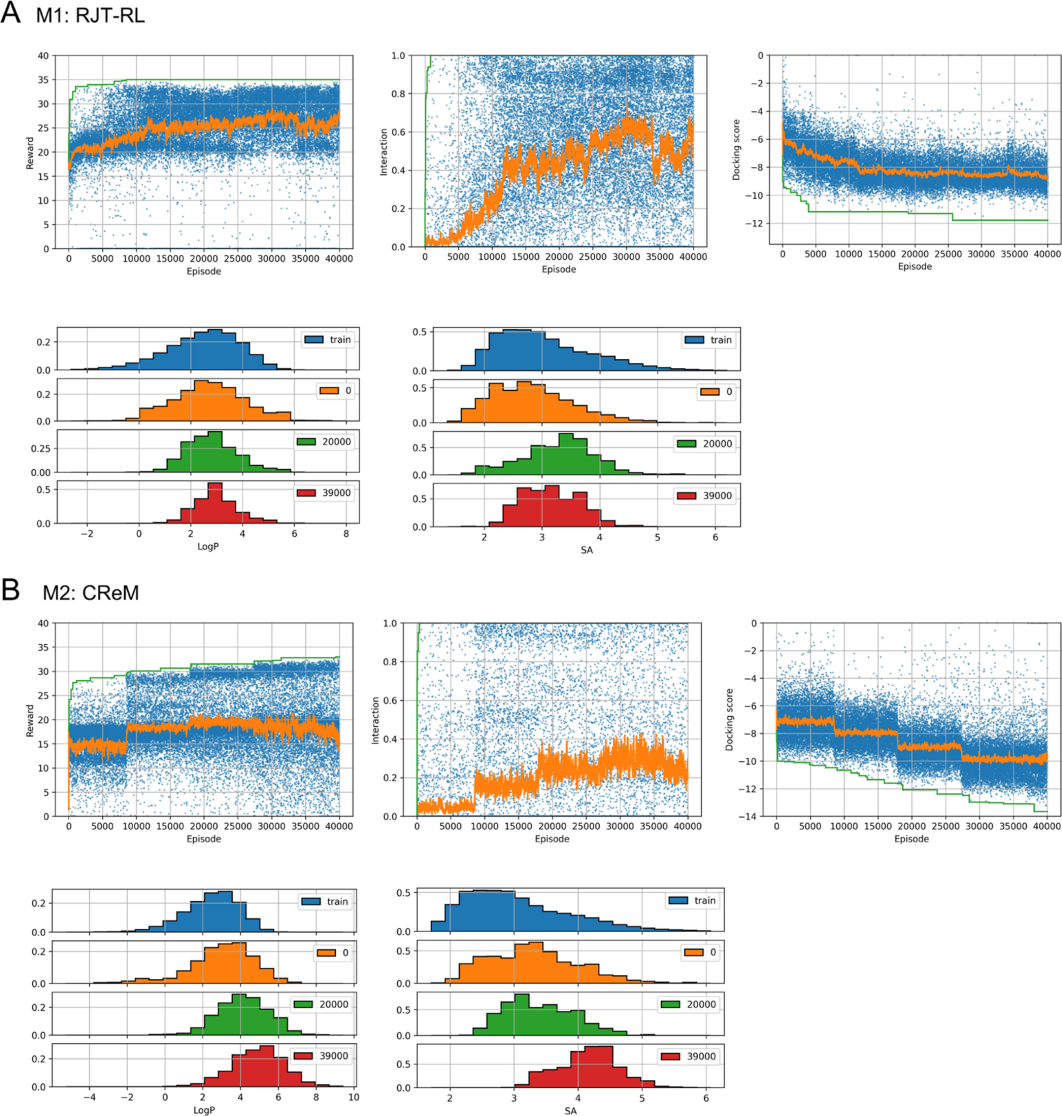
Results of the multiobjective reward experiments (A) M1 and (B) M2 (see Table 3). In the upper panel, the reward, interaction, and docking scores for each episode are plotted in blue, and the moving average and maximum values are plotted in orange and green, respectively. In the lower panel, histograms of the Log *P* and SA score distributions of the training dataset (blue) and episodes 0–1000 (orange), 20,000–21,000 (green), and 39,000–40,000 (red) are plotted.

**Figure 9:**
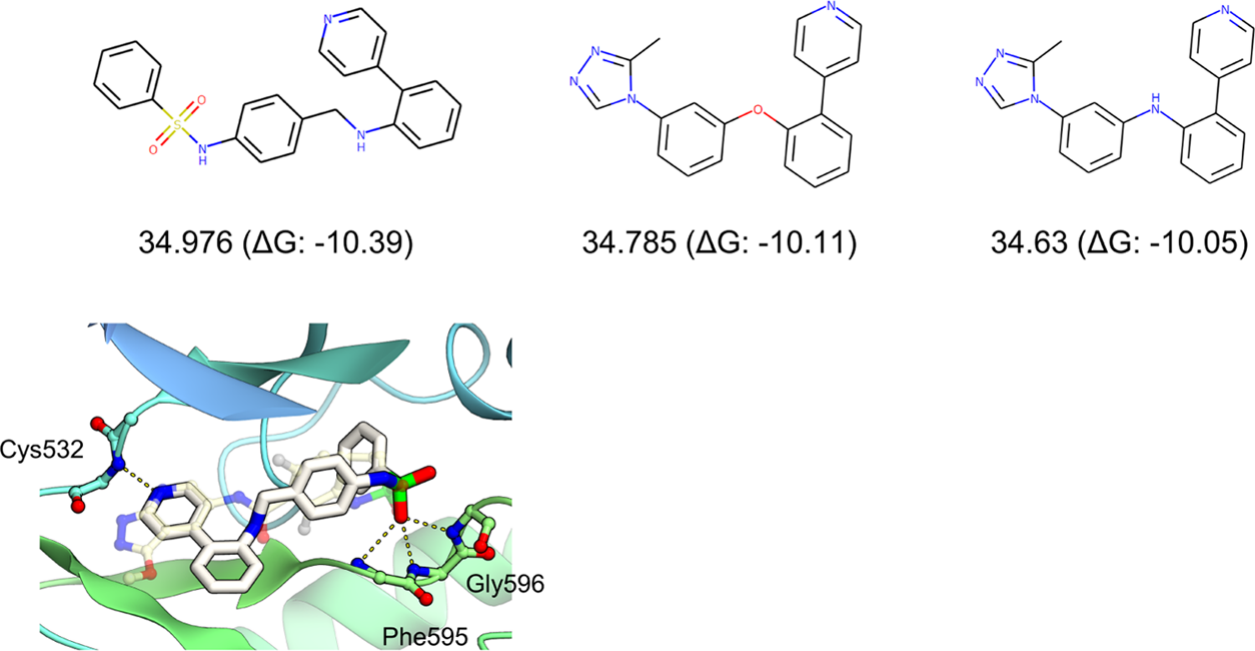
Compounds were generated in a multiobjective reward experiment, M1 (see Table 3). The chemical structures of the top three compounds, with their rewards and docking scores, are shown in the top panels. The binding poses of the best compounds are shown in the bottom panel. The protein backbone and bound inhibitor are shown using the ribbon and transparent ball-and-stick models, respectively. The original compound bound to the crystal structure is shown as semitransparent. The possible hydrogen-bonding interactions are indicated by yellow dashed lines.

The result of M1 demonstrated that the multiobjective reward function increased similar to the cases D1–D3, and the distributions of SA and Log *P* values were restricted to the specified ranges (Figures 8A and S11). In D1, molecules with high SA scores (SA > 4) were frequently generated (Figure S8A), whereas the generation of such molecules was suppressed in M1 (Figure 8A). The Log *P* value of the generated molecules was also restricted to the specified range compared to those in the D1 experiments (Figures 8A *vs*S8A). Consequently, the “filter score” in M1 was drastically improved compared to that of D1 (Table 3). The top three compounds in terms of reward and docking pose of the best compound are shown in Figure 9. Although the second and third compounds in Figure 9 are similar, the diversity of the generated structures of M1 can be observed from their statistics and plots (Table 3 and Figure S11). Collectively, these results demonstrate that RJT-RL can be successfully applied to multiobjective optimization. Moreover, another run of M1 was performed, and a similar tendency in the property distributions was observed (Figure S12). A similarity plot between these runs (Figure S12D) demonstrated that diverse molecules were generated from the different runs in this case.

Next, the results for RJT-RL (M1) and CReM (M2) were compared. CReM also successfully improved the overall score (Figure 8B). However, an unbalanced optimization of the score terms was observed, similar to the case of D4 (Figure 7D). Although the docking score was improved by optimization, the other terms (*i.e*., interaction, SA, and Log *P* terms) were less improved than those of M1 (Figure 8). Even though the increasing tendency of the SA score was suppressed, a large number of compounds with SA > 4.0 were still generated (Figure 8B). As for the Log *P* property, D4 generated more molecules with Log *P* in the specified range (0 < Log *P* < 5) than M2 (Figures S8D and 8B). To perform multiobjective optimization with CReM, we may have to perform an intensive hyperparameter search for the best weighting terms (*c*_intr_, *c*_SA_, and *c*_LogP_) in the scoring function and/or to introduce another penalty term for large molecules.

### Fine-Tuning to Different Reward Functions

One of the advantages of learning-based methods is their transferability to various tasks. To assess the transferability of the RJT-RL models (policy and value function), a fine-tuning experiment, F1, using different reward functions (Table 3) was performed. In this experiment, we attempted fine-tuning the model trained in D1 to the multiobjective reward function defined in M1.

**Figure 10:**
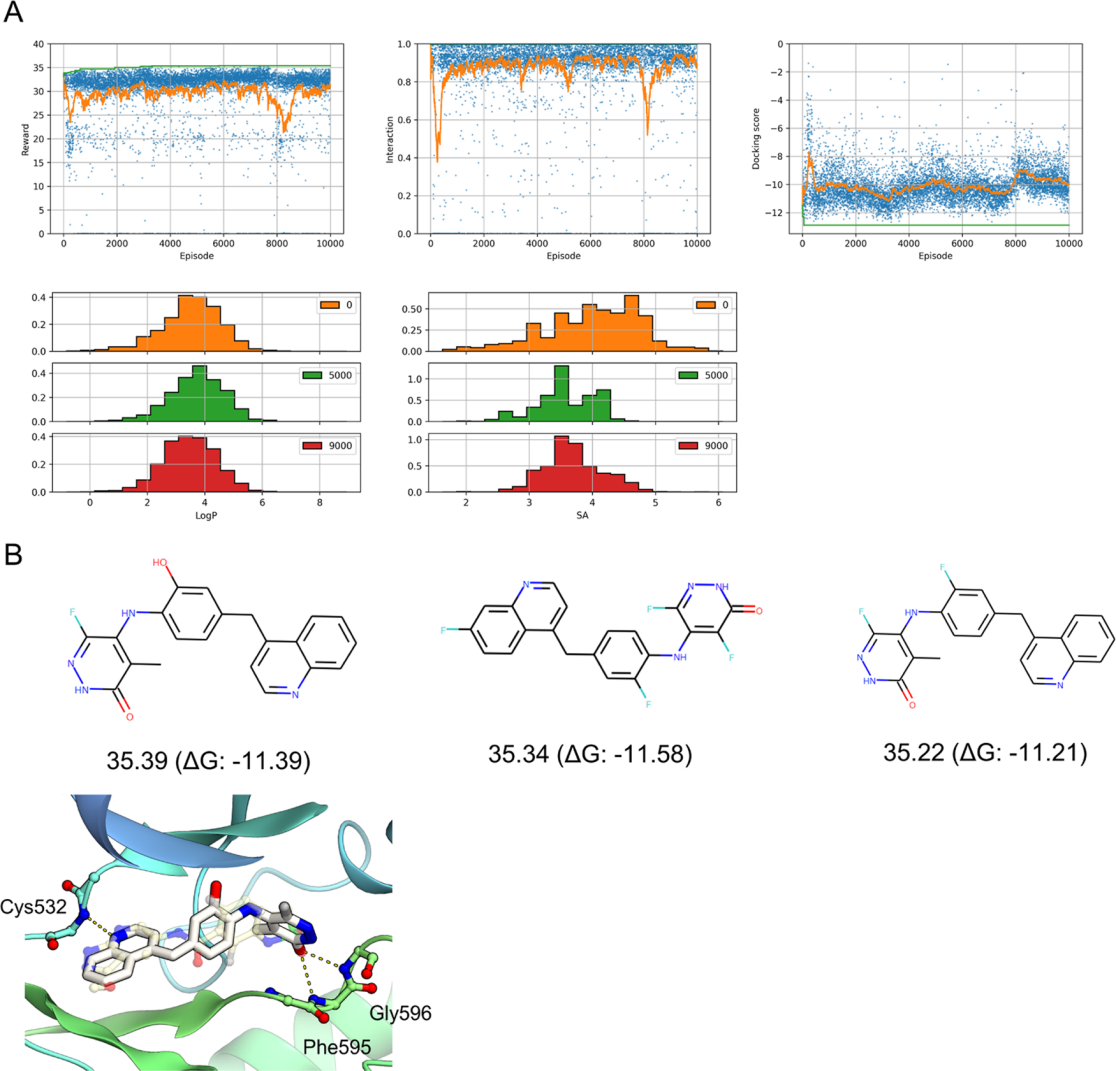
Results of fine-tuning experiment F1 (see Table 3). (A) In the upper panel, the reward, interaction, and docking scores for each episode are plotted in blue, and the moving average and maximum values are plotted in orange and green, respectively. In the lower panel, histograms of the Log *P* and SA score distributions of episodes 0–1000 (orange), 5000–6000 (green), and 9000–10,000 (red) are plotted. (B) Chemical structures of the top three compounds with their rewards and docking scores are shown in the top panels. The binding poses of the best compounds are shown in the bottom panel as in Figure 9.

The results of F1 are shown in Figures 10 and S13. From the episodes in the early training stage, the agent generated molecules with high docking and interaction scores, which were maintained during the training (Figure 10). The SA and Log *P* scores also gradually improved as the training proceeded, and their distributions fell within the range specified by the penalty score (*i.e*., SA < 4 and 0 < Log *P* < 5) (Figure 10). The filter score of F1 was improved from that of D1 (Table 3), suggesting that the distribution of the generated compounds contained more feasible structures than before fine-tuning. In contrast, the diversity of molecules generated by F1 was slightly lower than that of M1, as evident from their statistics (uniqueness and IntDev in Table 3). The top three compounds in terms of reward and the docking pose of the best compound are shown in Figure 10. This result demonstrates that the agent adapted to the different reward functions containing the penalty terms after fewer training steps than those in M1.

## Conclusions

In this study, we introduced RJT, a coarse-grained representation of molecules, which is directly convertible to the original chemical structure. We leveraged this representation for drug discovery, formulating a molecule design task as reinforcement learning to generate an RJT. The proposed method exhibited a better or comparable performance to other state-of-the-art methods in simple benchmark tasks. The results indicate the potential of the step-by-step evaluation of molecular properties, which is only possible in our framework using RJT. The step reward was shown to be advantageous when the computational cost of the score calculation was low because the score required multiple evaluations to generate a single compound. We further demonstrated that our method is applicable to real-world tasks such as structure-based scaffold hopping with a multiobjective reward function. Another advantage of RTJ-RL is the fine-tuning of the policy and value function models, which is not possible with rule-based fragment growth methods. This feature may be useful for tuning scoring functions in the compound design process.

The results of the experiments also suggest several problems with the proposed method. For example, considering the 3D generation involving docking simulation, efficient training of the RJT-RL model requires proper handling of the stereoisomers of the generated compounds. In this study, we enumerated the possible stereoisomers and searched for the best stereoisomers using the brute-force method. However, the search was performed on a single CPU, which reduced the overall performance (Table 5). Parallelization of the 3D conformer generation and docking simulation could improve performance because this calculation is embarrassingly parallelizable. Another solution to this problem is to extend the site information in the RJT representation to properly handle the chirality flag of the atom so that the agent can generate the molecule, including the stereoisomers. Finally, in this study, we only considered the de novo design of compounds from a relatively small starting fragment size. To apply the proposed method for the optimization of lead compounds with larger and more complex structures, it is necessary to extend the action to include node deletion and mutation to enable the modification of the starting scaffolds.

## Notes

The materials supporting this article, including the source code, sampled molecules, and pretrained models, are available at github (https://github.com/pfnet-research/RJT-RL) under the MIT license.
