# Supplementary material for: Molecular Design Method Using a Reversible Tree Representation of Chemical Compounds and Deep Reinforcement Learning
Source: J Chem Inf Model. 2022 Aug 12;62(17):4032–48. doi: 10.1021/acs.jcim.2c00366 (PMC9472278; doi:10.1021/acs.jcim.2c00366)
Supplement: Supplementary file 1 — ci2c00366_si_001.pdf [file ci2c00366_si_001.pdf]

Supporting information for:

Molecular design method using a reversible tree representation of chemical compounds and deep reinforcement learning

Ryuichiro Ishitani*, Toshiki Kataoka, Kentaro Rikimaru

Preferred Networks, Inc., Tokyo, Japan.

Supporting Information

Figure S1

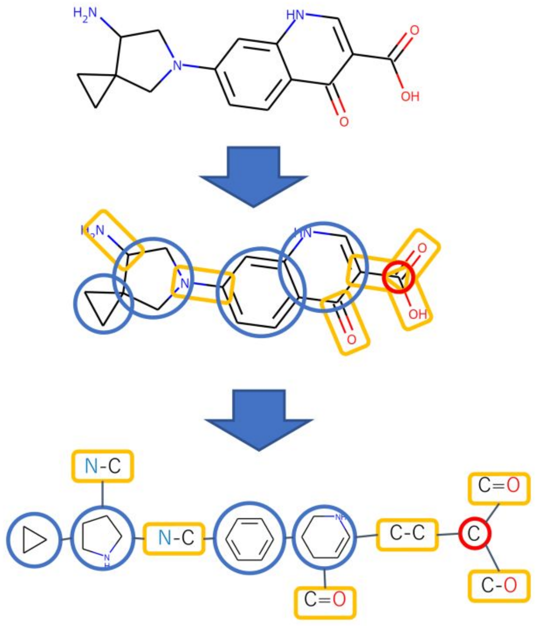

Schematic showing an example of the tree decomposition algorithm used in this study. A molecular graph $\mathcal{G}$ (upper row) is decomposed into fragments that are assigned to nodes (middle row). The nodes are then connected to form a tree structure $\mathcal{T}$ (bottom row). For a detailed description of the tree decomposition algorithm, please refer the literature[1].

Figure S2

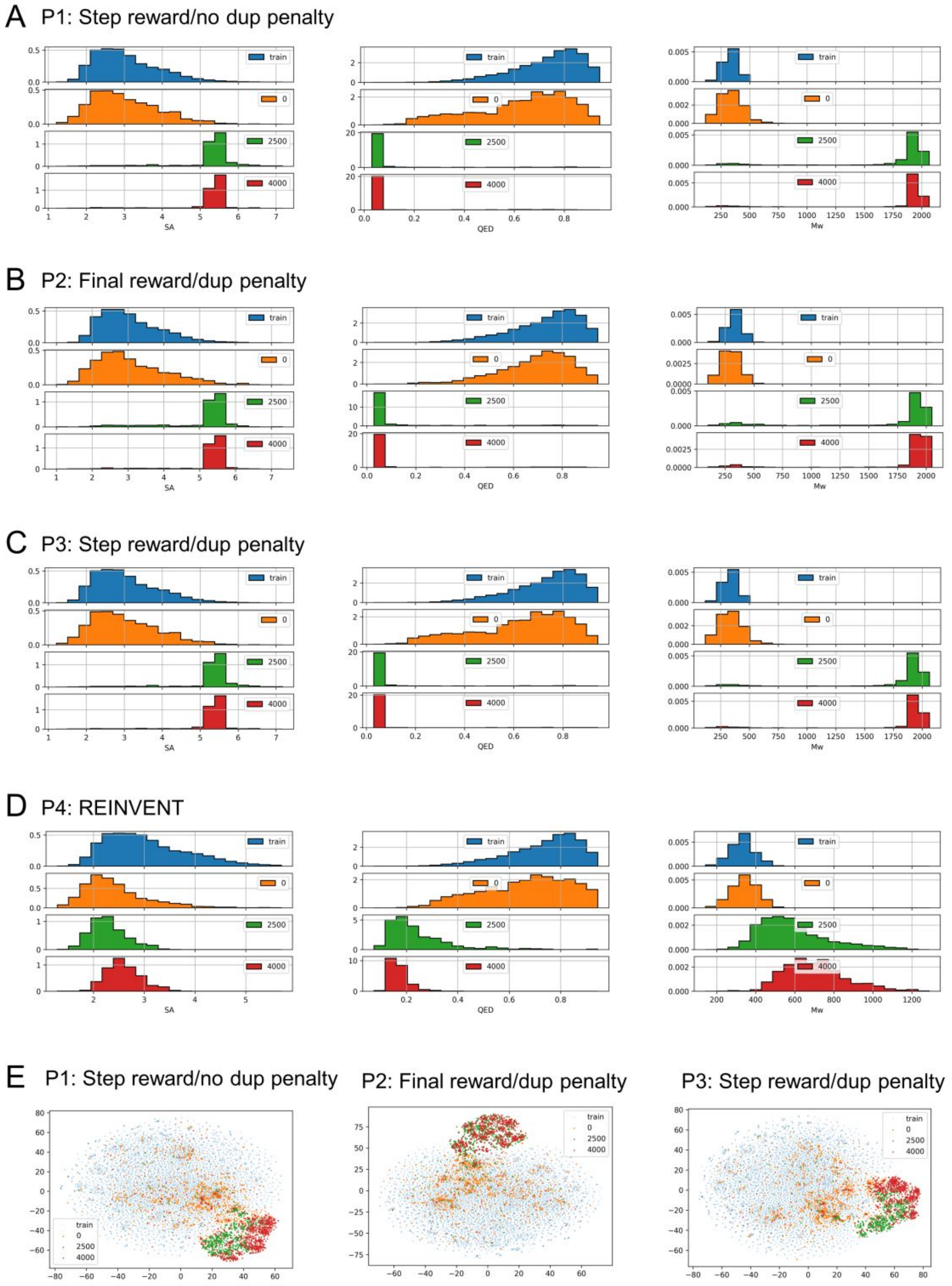

Property distributions of molecules generated by penalized-LogP experiments (P1–P4).

(A–D) SA and QED[2] (ref) scores and molecular weight distributions. The

distributions of the training dataset (blue) and episodes 0–1,000 (orange), 2,500–3,500 (green), and 4,000–5,000 (red) are plotted.

(E) Distributions of compounds from the training dataset (blue) and episodes 0–1,000 (orange), 2,500–3,500 (green), and 4,000–5,000 (red). The FCFP4 fingerprint vectors[3] of the molecules were projected onto a 2D space using the t-distributed stochastic neighbor embedding (t-SNE) algorithm[4]. For the training dataset, 10,000 molecules were randomly extracted.

Figure S3

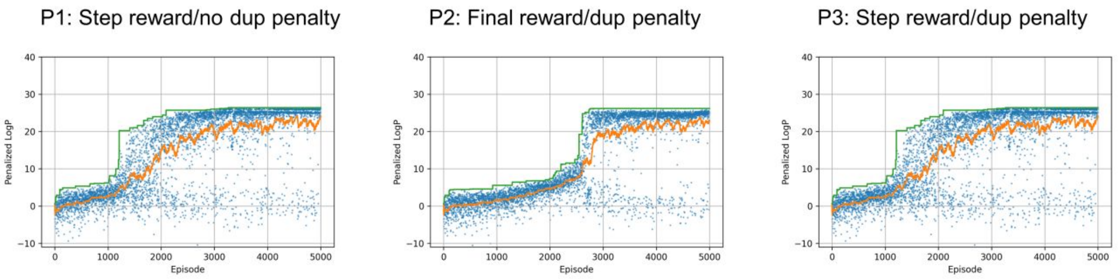

Other runs of the penalized LogP experiment. The penalized LogP values for each episode are plotted in green, whereas their moving average and maximum values are plotted in orange and green, respectively.

Figure S4

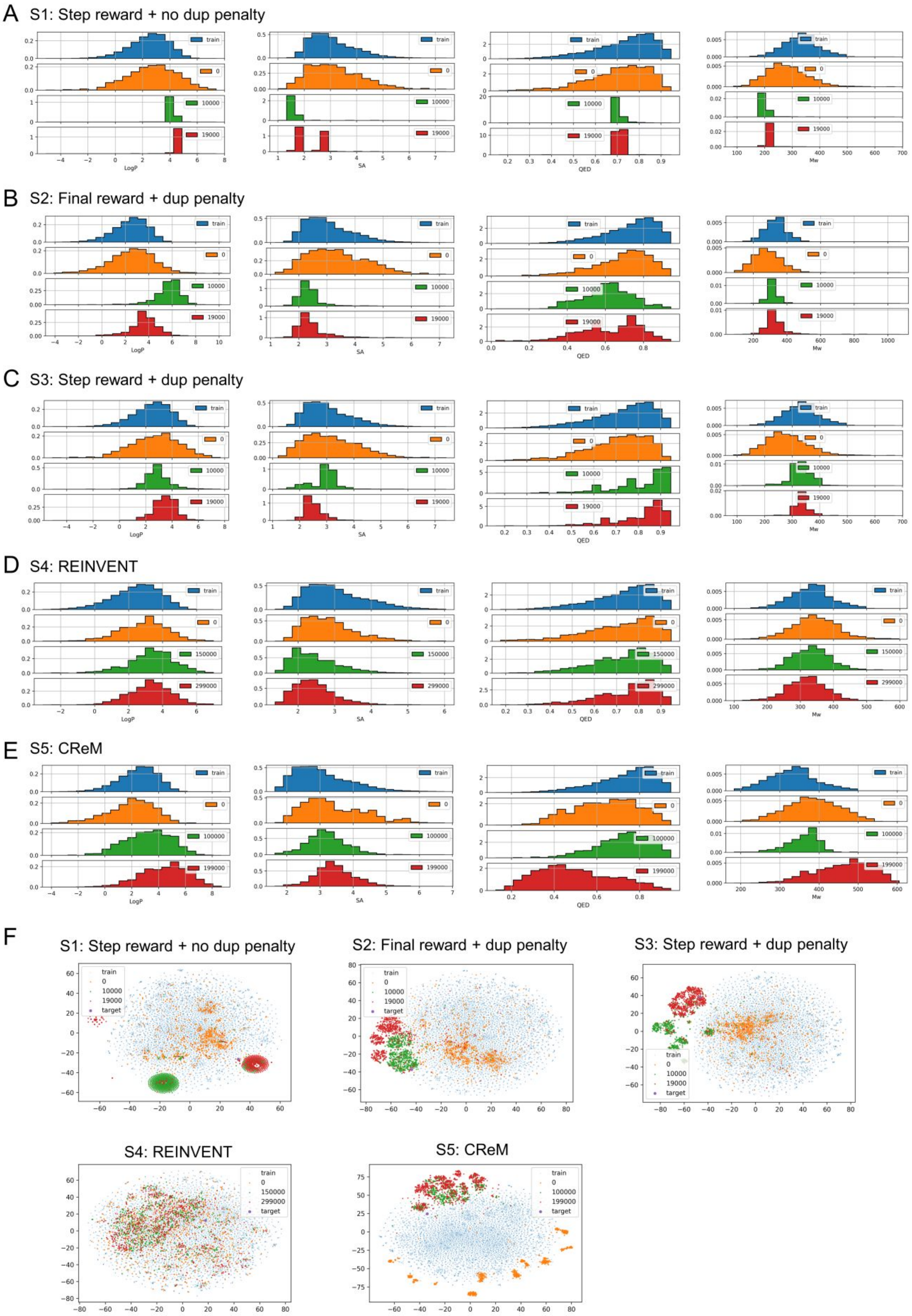
A S1: Step reward + no dup penalty
B S2: Final reward + dup penalty
C S3: Step reward + dup penalty
D S4: REINVENT
E S5: CReM
F
S1: Step reward + no dup penalty
S2: Final reward + dup penalty
S3: Step reward + dup penalty
S4: REINVENT
S5: CReM
train
0
10000
19000
150000
299000
100000
199000
target
LogP
SA
QED
Mw

Property distributions of the generated molecules by the vortioxetine similarity experiments (S1–S5).

(A–C) LogP, SA, and QED scores and molecular weight distributions in experiments S1–S3. Distributions of the training dataset (blue) and episodes 0–1,000 (orange), 10,000–11,000 (green), and 19,000–20,000 (red) are plotted.

(D) LogP, SA, and QED scores and molecular weight distributions in experiment S4. Distributions of the training dataset (blue) and episodes 0–1,000 (orange), 150,000–151,000 (green), and 299,000–300,000 (red) are plotted.

(E) LogP, SA, and QED scores and molecular weight distributions in experiment S5. Distributions of the training dataset (blue) and episodes 0–1,000 (orange), 100,000–101,000 (green), and 199,000–200,000 (red) are plotted.

(F) Distributions of compounds from the training dataset (blue), episodes 0–1,000 (orange), 10,000–11,000 (green), and 19,000–20,000 (red), and vortioxetine (purple). The distributions were calculated as per Fig. S2E.

Figure S5

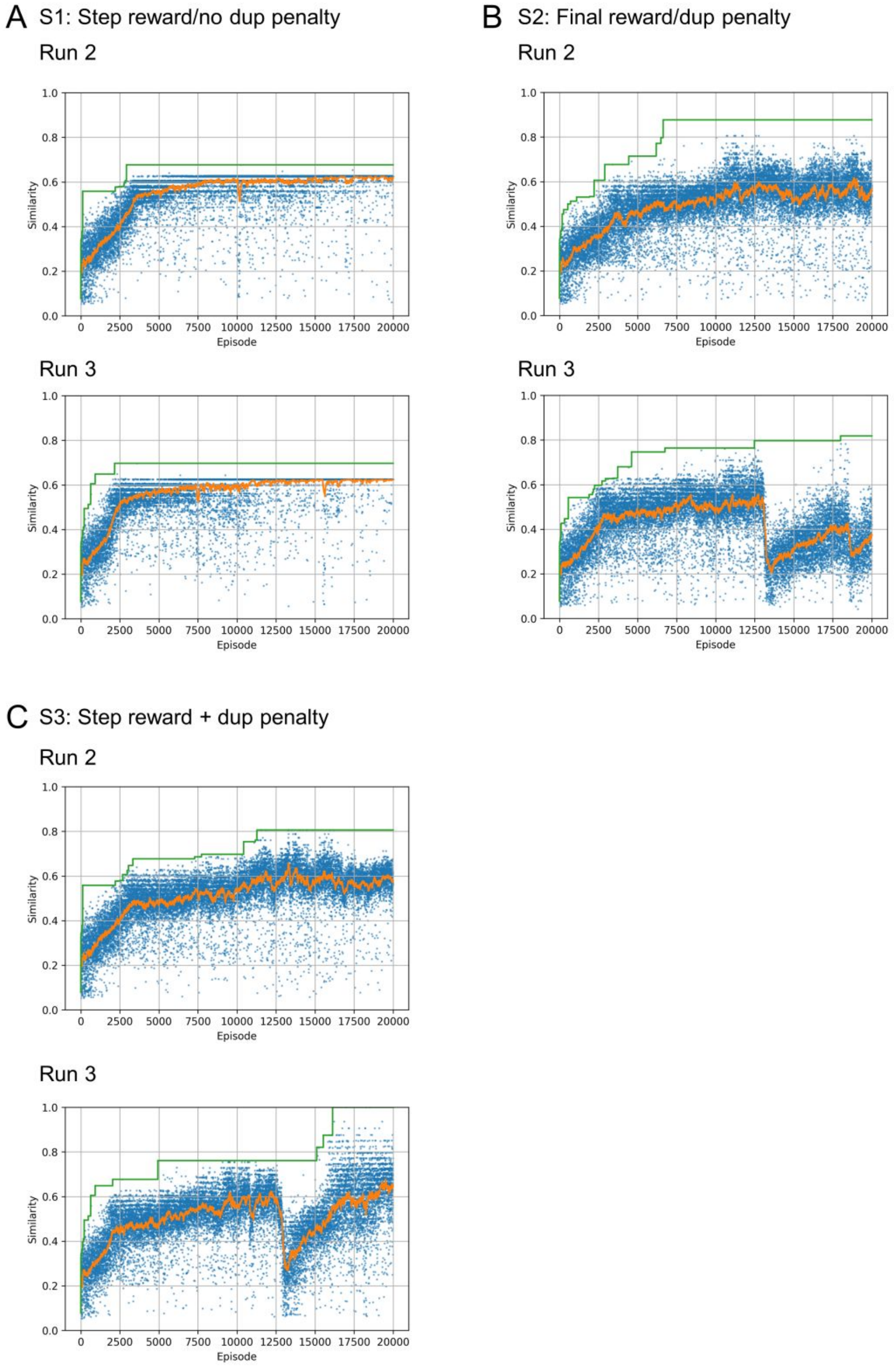

Other runs of the vortioxetine similarity experiments. For (A) S1, (B) S2, and (C) S3, the similarity scores are plotted in green, whereas their moving averages and maximum values are plotted in orange and green, respectively.

Figure S6

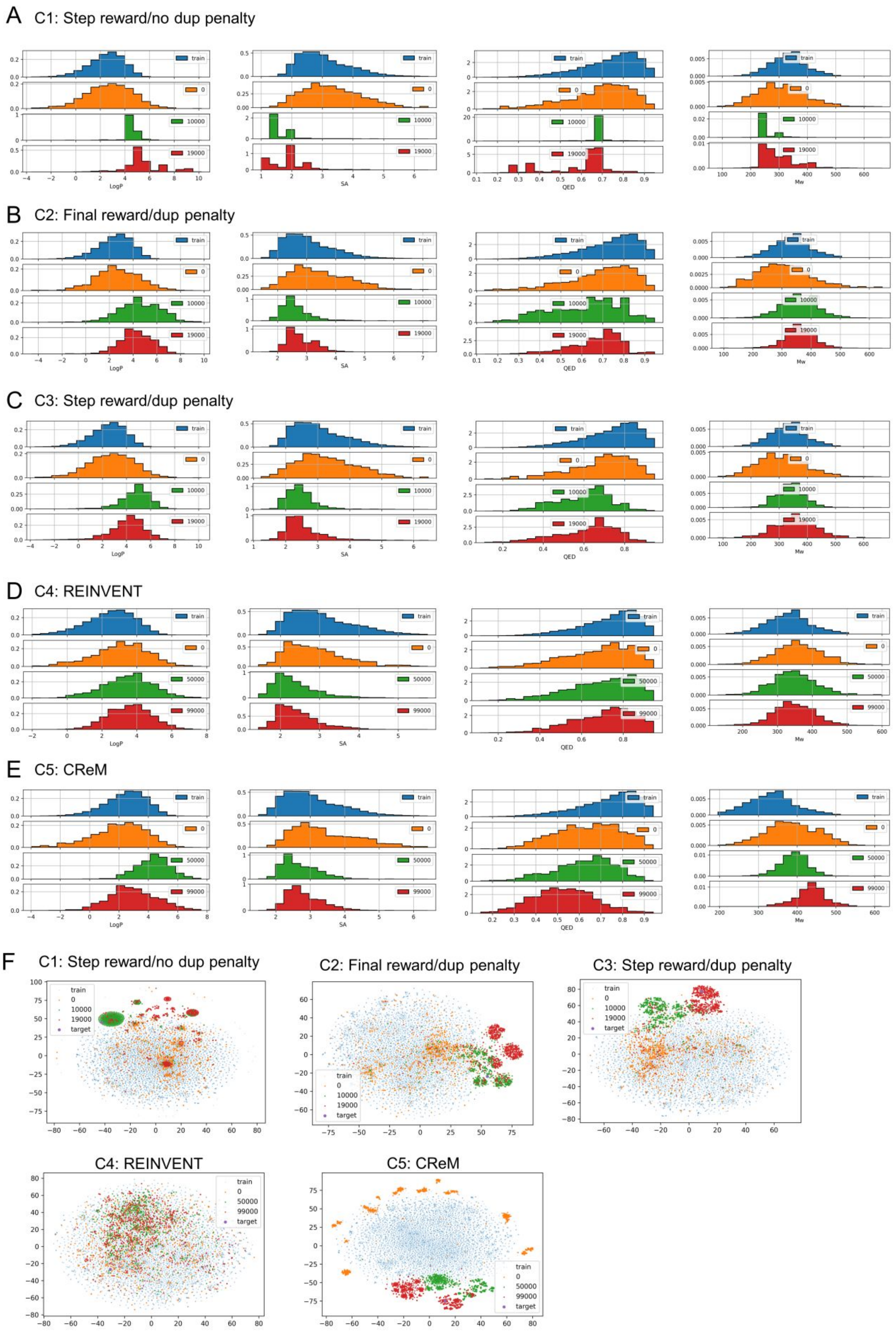
A
C1: Step reward/no dup penalty
train
0
10000
19000
LogP
SA
QED
Mw
B
C2: Final reward/dup penalty
train
0
10000
19000
LogP
SA
QED
Mw
C
C3: Step reward/dup penalty
train
0
10000
19000
LogP
SA
QED
Mw
D
C4: REINVENT
train
0
50000
99000
LogP
SA
QED
Mw
E
C5: CReM
train
0
50000
99000
LogP
SA
QED
Mw
F
C1: Step reward/no dup penalty
C2: Final reward/dup penalty
C3: Step reward/dup penalty
train
0
10000
19000
target
C4: REINVENT
C5: CReM
train
0
50000
99000
target

Property distributions of the generated molecules by the celecoxib similarity experiments (C1–C5).

(A–C) LogP, SA, and QED scores and molecular weight distributions in experiments C1–C3. Distributions of the training dataset (blue) and episodes 0–1,000 (orange), 10,000–11,000 (green), and 19,000–20,000 (red) are plotted.

(D–E) LogP, SA, and QED scores and molecular weight distributions in experiments C4 and C5. Distributions of the training dataset (blue) and episodes 0–1,000 (orange), 50,000–51,000 (green), and 99,000–100,000 (red) are plotted.

(F) Distributions of compounds from the training dataset (blue), episodes 0–1,000 (orange), 10,000–11,000 (green), and 19,000–20,000 (red), and celecoxib (purple). The distributions were calculated as per Fig. S2E.

Figure S7

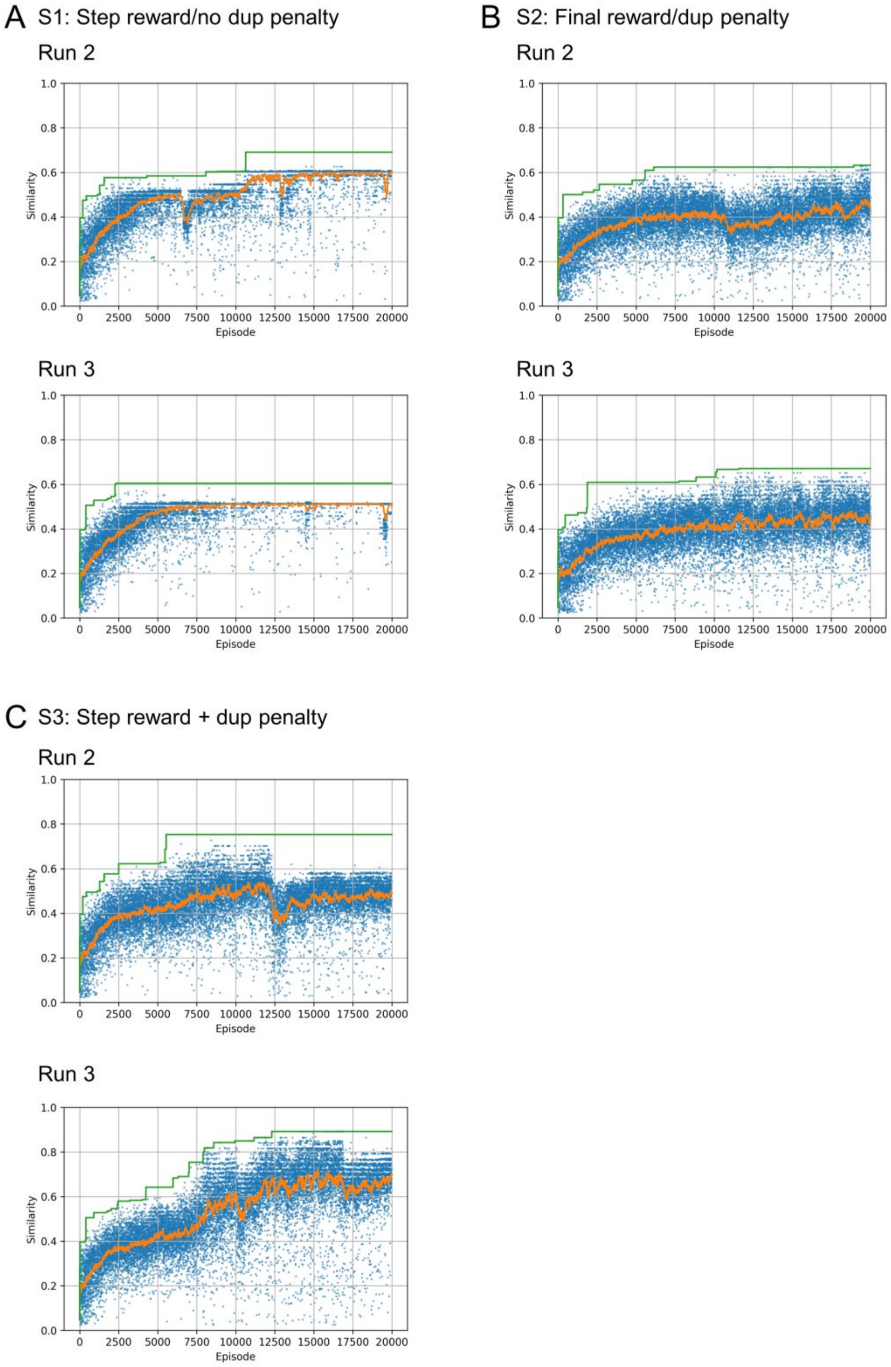

Other runs of the celecoxib similarity experiments. For experiments (A) C1, (B) C2, and (C) C3, the similarity scores are plotted in green, whereas their moving averages and maximum values are plotted in orange and green, respectively.

Figure S8

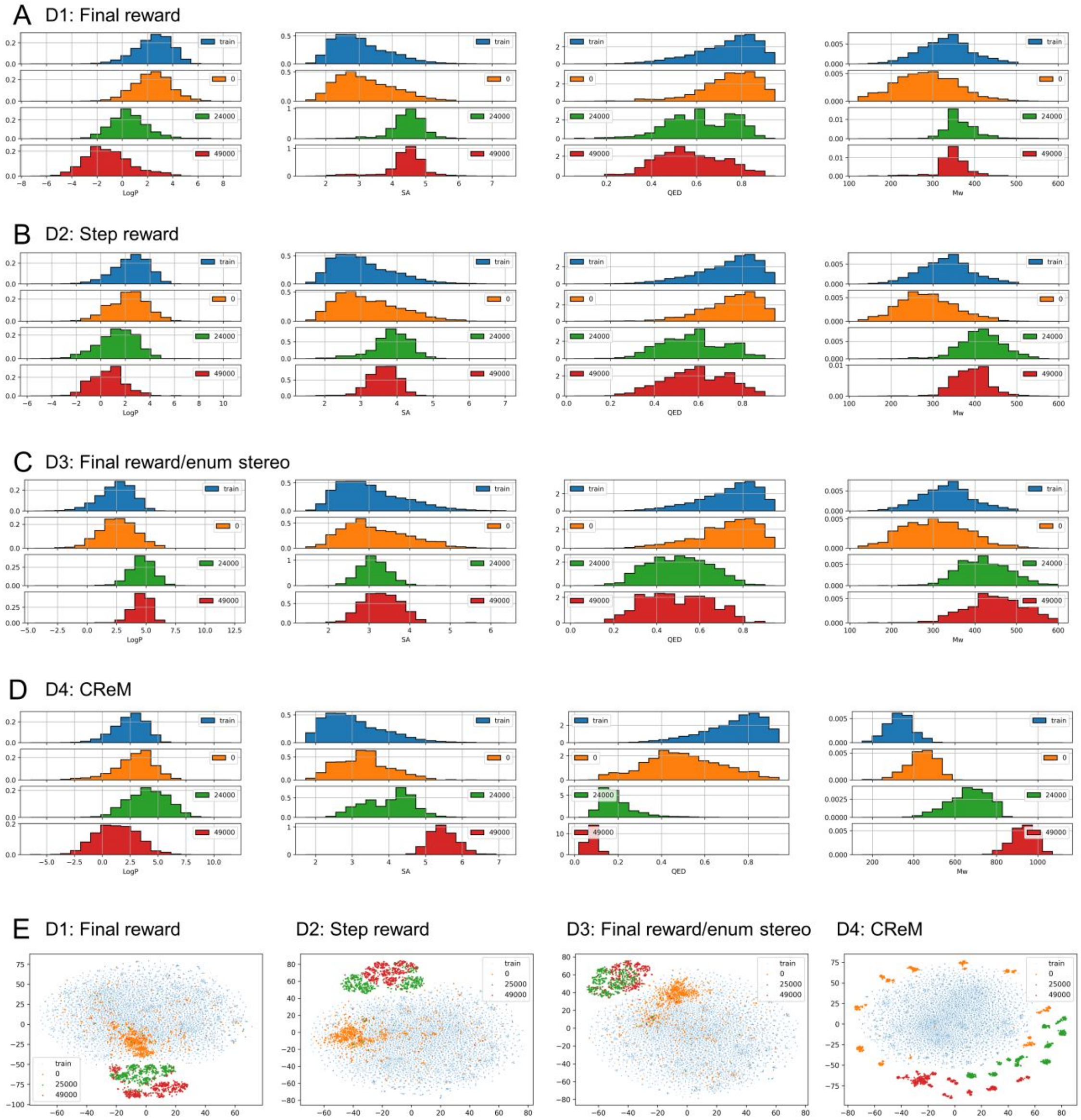

Property distributions of the generated molecules by the docking experiments (D1–D4).

(A–D) LogP, SA, and QED scores and molecular weight distributions in experiments C1–C3. Distributions of the training dataset (blue) and episodes 0–1,000 (orange), 24,000–25,000 (green), and 49,000–50,000 (red) are plotted.

(F) Distributions of compounds from the training dataset (blue) and episodes 0–

1,000 (orange), 25,000–26,000 (green), and 49,000–50,000 (red). The fingerprint distributions were calculated as per Fig. S2E.

Figure S9

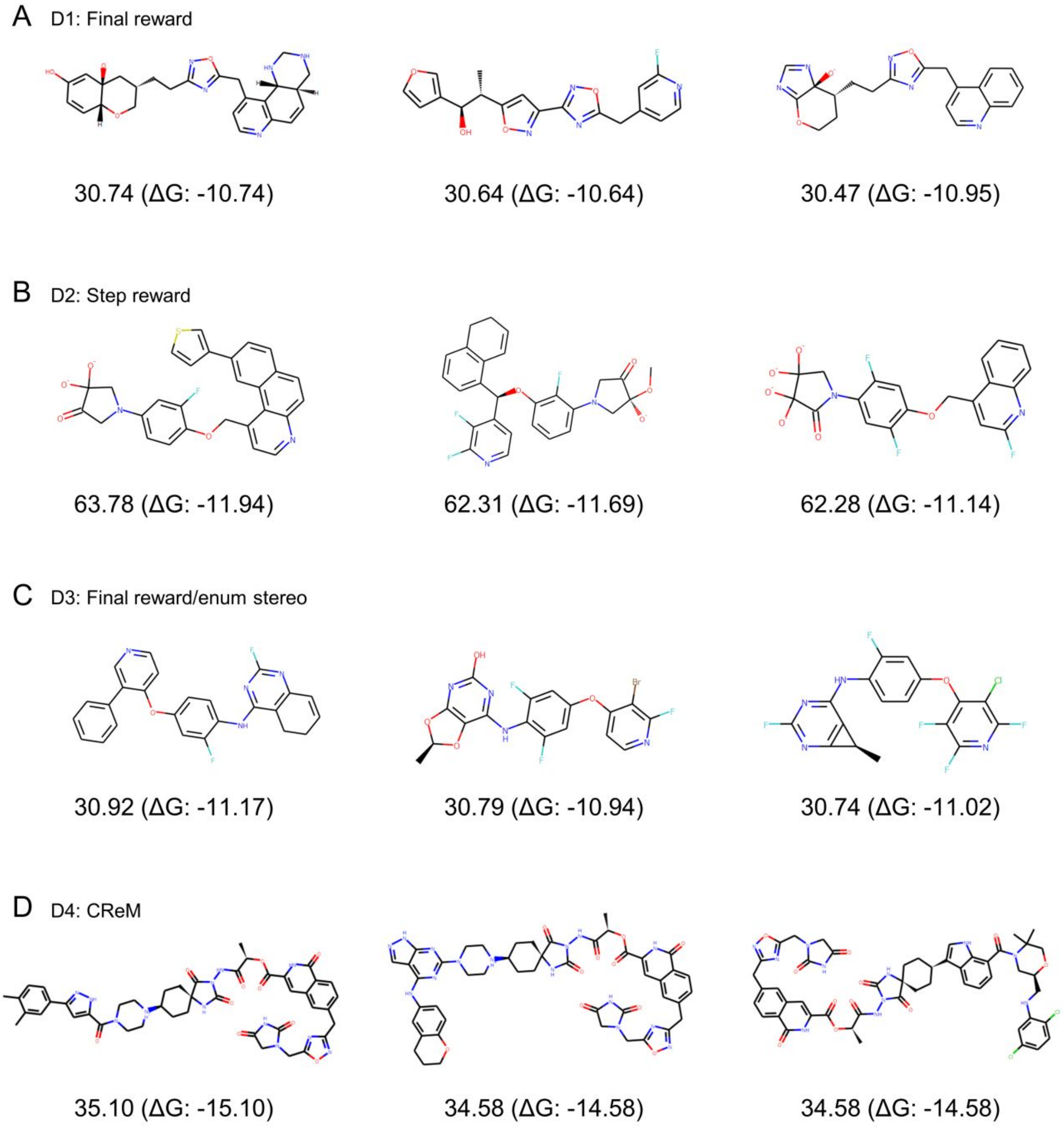

Chemical structures of the molecules generated by docking experiments (A) D1, (B) D2, (C) D3, and (D) D4. The rewards and docking scores are presented below:

Figure S10

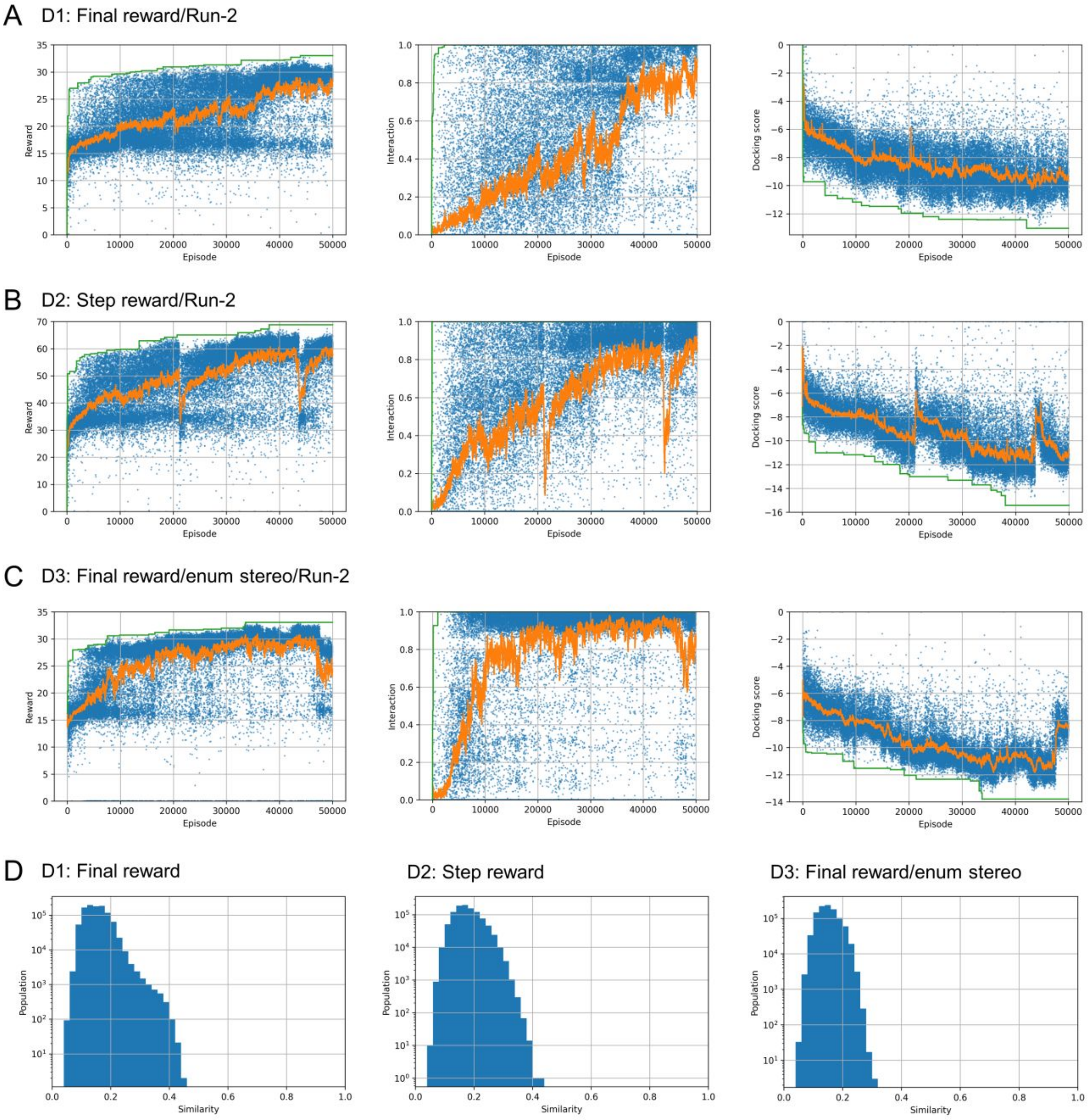

Results of other docking experiment runs. For (A) D1, (B) D2, and (C) D3, the reward (left panel), interaction score (middle panel), and docking score (left panel) are plotted in green, whereas their moving average and maximum values are plotted in orange and green, respectively.

(D) Distributions of pairwise similarities between the top-1000 molecules from two different runs. The histograms of $T(m_1,m_2), m_1 \in \mathcal{M}_1, m_1 \in \mathcal{M}_2$ are plotted, where

$T(i,j)$ is the Jaccard index[5] of the FCFP4 fingerprints[3] of molecules i and j, and $\mathcal{M}_1$ and $\mathcal{M}_2$ are the sets of the top-1000 molecules from two different runs.

Figure S11

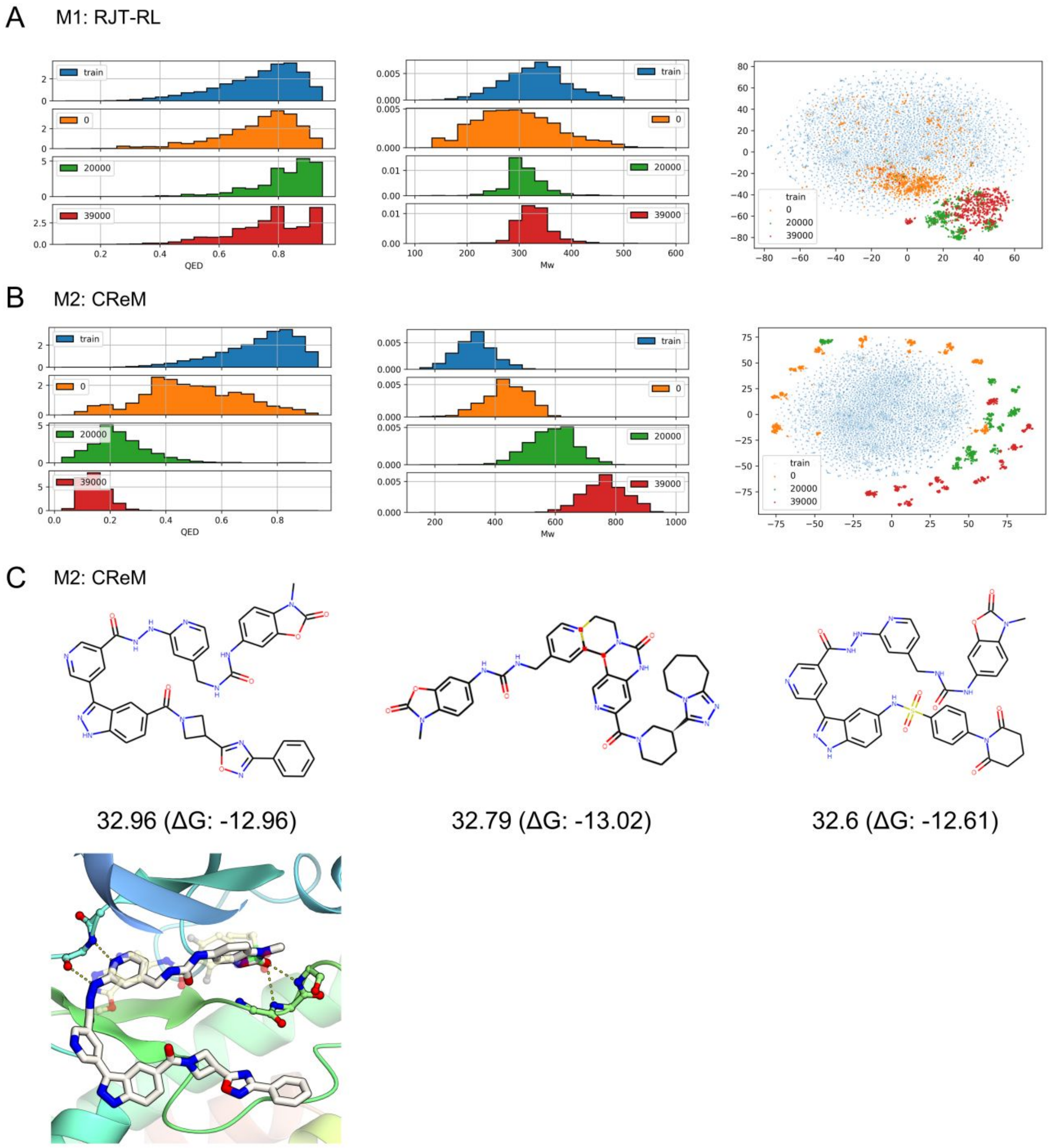

Results of the multi-objective reward experiments, M1 and M2 (see Table 3 in the main text).

(A) and (B) QED scores (left panel), molecular weights (middle panel), and fingerprint distributions (right panel) for experiments M1 and M2, respectively. The distributions of the training dataset (blue) and episodes 0–1,000 (orange), 20,000–21,000 (green), and 39,000–40,000 (red) are plotted. The fingerprint distribution was calculated

as per Fig. S2E.

(C) Compounds generated by experiment M2 (see Table 3 in the main text). The chemical structures of the top three compounds, with their rewards and docking scores, are shown in the top panels. The binding poses of the best compounds are shown in the bottom panel.

Figure S12

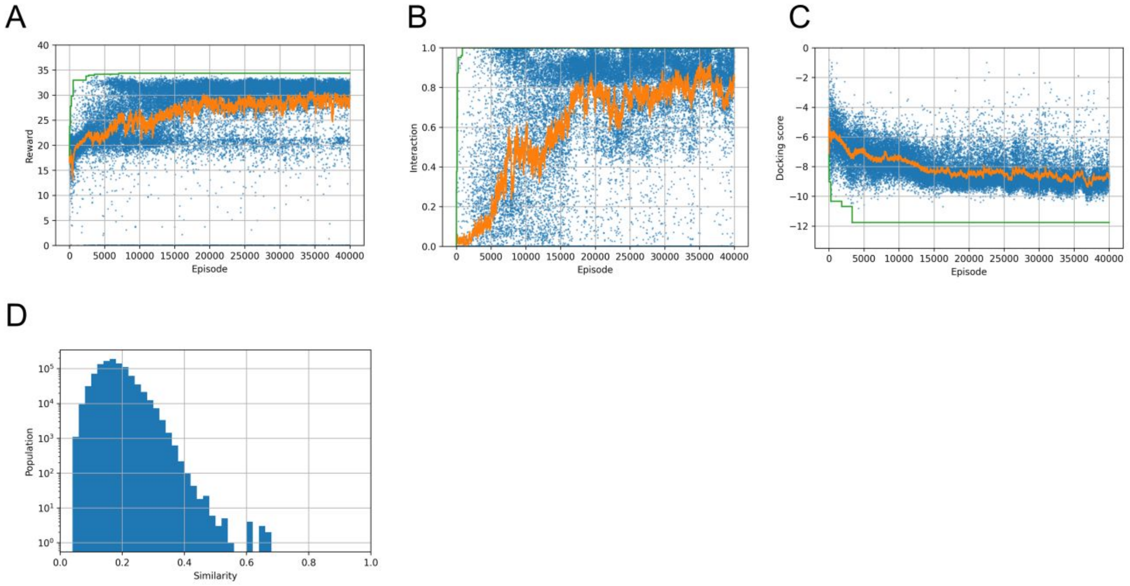

Results of other multi-objective reward experiment runs. (A) The reward, (B) interaction score, and (C) docking score are plotted in green, and their moving average and maximum values are plotted in orange and green, respectively. (D) Distribution of pairwise similarity between the top-1000 molecules from two different runs. The histogram was calculated as per Fig. S10D.

Figure S13

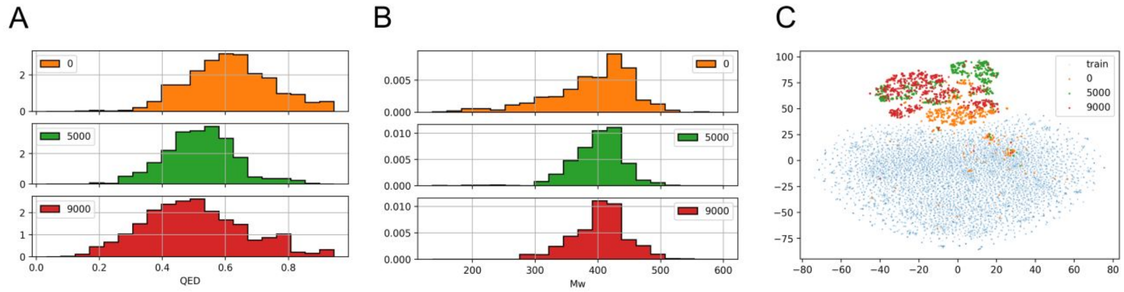

Results of the fine-tuning experiment F1 (see Table 3 in the main text). The QED score (A), molecular weight (B), and fingerprint distributions (C) for the training dataset (blue) and episodes of 0–1,000 (orange), 20,000–21,000 (green), and 39,000–40,000 (red) are plotted. The fingerprint distribution was calculated as per Fig. S2E.

References

(1) Jin W, Barzilay R, Jaakkola T (2018) Junction Tree Variational Autoencoder for Molecular Graph Generation. arXiv 1802.04364 https://doi.org/10.48550/arXiv.1802.04364

(2) Bickerton G R, Paolini G v, Besnard J, Muresan S, Hopkins A L (2012) Quantifying the Chemical Beauty of Drugs. Nat Chem 4:90–98 https://doi.org/10.1038/nchem.1243

(3) Rogers D, Hahn M (2010) Extended-Connectivity Fingerprints. J Chem Inf Model 50:742–754 https://doi.org/10.1021/ci100050t

(4) der Maaten L, Hinton G (2008) Visualizing Data Using T-SNE. J Mach Learn Res 9 2579–2605

(5) Jaccard P (1901) Étude Comparative de La Distribution Florale Dans Une Portion Des Alpes et Des Jura. Bull Soc Vaud Sci Nat 37:547–579
